# The effects of the Nepal community forestry program on biodiversity conservation and carbon storage

**DOI:** 10.1371/journal.pone.0199526

**Published:** 2018-06-26

**Authors:** Harisharan Luintel, Randall A. Bluffstone, Robert M. Scheller

**Affiliations:** 1 SmartStart Research and Evaluation, Irvine, California, United States of America; 2 ForestAction Nepal, Kathmandu, Nepal; 3 Department of Economics, Portland State University, Portland, Oregon, United States of America; 4 Department of Forestry and Environmental Resources, North Carolina State University, Raleigh, North Carolina, United States of America; Sichuan University, CHINA

## Abstract

Approximately 15.5% of global forest is controlled by ~1 billion local people and the area under community control is increasing. However, there is limited empirical evidence as to whether community control is effective in providing critical global ecosystem services, such as biodiversity conservation and carbon storage. We assess the effectiveness of one example of community-controlled forest, Nepal’s Community Forestry Program (CFP), at providing biodiversity conservation and carbon storage. Using data from 620 randomly selected CFP and non-CFP forest plots, we apply a robust matching method based on covariates to estimate whether CFPs are associated with greater biodiversity conservation or carbon storage. Our results reveal a significant positive effect of CFP on biodiversity, which is robust against the influence of unobserved covariates. Our results also suggest a significant negative effect of the CFP on aboveground tree and sapling carbon (AGC) at the national scale (-15.11 Mg C ha^-1^). However, the CFP has a mixed effect on carbon across geographic and topographic regions and in forests with different canopy covers. Though there were no significant effects of the CFP on AGC at lower altitudes, in the Terai or hill regions, and under closed canopies, there were positive effects in open canopies (25.84 Mg C ha^-1^) at lower slopes (25.51 Mg C ha^-1^) and negative effects at higher altitudes (-22.81 Mg C ha^-1^) and higher slopes (-17.72 Mg C ha^-1^). Our sensitivity analysis revealed that the positive effects are robust to unobserved covariates, which is not true for the negative results. In aggregate, our results demonstrate that CFP can be an effective forest management strategy to contribute to global ecosystem services such as biodiversity, and to a lesser extent carbon.

## Introduction

Over one billion people control ~15.5% of global forests, and the area under community control is increasing [1]. Expecting positive effects on forest health, local ecosystem services and communities’ livelihoods, during the past 40 years governments in many tropical countries have transferred forest rights to local communities [2, 3]. Recently, global environmental initiatives, such as the Convention on Biological Diversity (CBD) and Reducing Emissions from Deforestation and Forest Degradation (REDD+) recognize forest devolution and decentralization as a vehicle for biodiversity conservation and carbon storage. Such initiatives primarily focus on tropical forests, as they host 34 global biodiversity hot spots [4], store 40% of terrestrial carbon and emit 17% of anthropogenic greenhouse gas emissions [5].

The community forestry program (CFP) in Nepal is part of a worldwide trend toward forest devolution started four decades ago, which provides legal opportunities for local communities to manage and use forest resources [6]. This devolution has been recognized as a major accomplishment in natural resource management and is often credited with successfully curbing deforestation, revitalizing degraded forests and protecting forests, while supporting local livelihoods [7, 8, 9, 10]. Recent studies suggest that it could also increase carbon storage [11, 12, 13].

But naive implementation of forest devolution programs, including the CFP, may not guarantee more biodiversity conservation and/or carbon storage. A systematic review demonstrated that there is no evidence that community forest management programs are associated with global environmental benefits, with the exception of tree density and basal area [14]. Advocates pitch decentralized forestry as an effective approach to increase forest ecosystem services, such as fuelwood, fodder, and water quality, that improve human wellbeing [15, 1]. The reasons for devolving forests, including in Nepal, generally therefore have little to do with biodiversity and carbon *per se* [16] and better community rights may instead increase extractions of biomass and reduce biodiversity and/or carbon [17].

For example, early work by Flint & Richards [18], examined the effect of land use change in South Asia on carbon sequestration. They found that harvesting of fuelwood and timber, grazing, and burning of fields by local communities in some cases resulted in loss of forest carbon. Furthermore, though communities often do not actively manage forests [19], when forests are actively managed, communities may carry out species-preferred silvicultural practices, potentially leading to reduced plant species diversity [20].

Though small-scale, localized research has been conducted; there are few rigorous studies at larger scales that have evaluated the effects of community forestry on biodiversity and carbon. Bottazoi et al. [21] focused on the Bolivian Amazon and determined that different production structures across communities yield widely varying outcomes for carbon and biodiversity. Using worldwide data, Chhatre and Agrawal [22] estimated the effect of collective action on carbon sequestration and community livelihoods; they found that tradeoffs and synergies are possible. Beyene et al. [23] estimated the effect of local community forestry collective action on carbon storage in Ethiopia, but found minor effects. Using the same data as in this paper, Bluffstone et al. [24] found mixed effects of three measures of collective action on carbon storage and three other forest quality measures.

For this research, we estimate whether and how much Nepal’s CFP affects plant species diversity and carbon storage. We used 2013 cross-sectional data from a nationally representative random sample of CFP and corresponding non-CFP communities and forests. A key challenge in evaluating the influence of CFPs is deriving an appropriate counterfactual condition that allows consideration of what would have happened in the absence of the program. As the CFP areas are unlikely to be randomly distributed across the country, overcoming potential selection bias was critical. We therefore used a quasi-experimental matching approach through which we identified a counterfactual control group from non-CFP areas; this approach provided insights into what would have happened to biodiversity and carbon in the absence of the CFP.

To implement this matching, we identified confounders affecting the assignment of forests into the CFP and controlled for them through a matching process that seeks to mimic randomized experiments [25]. Using only matched observations, we estimated the average effect of the CFP on biodiversity (ATT_B_) and carbon (ATT_C_), measured as effective number of species (e^H’^) and aboveground tree and sapling carbon (AGC). As national-level estimates may mask a great deal of variation in the effectiveness of the CFP, we estimated ATT_B_ and ATT_C_ for various geographic, topographic, and geo-political regions and forest types. We also explored the extent to which our ATT_B_ and ATT_C_ estimates were robust, by testing their sensitivity to bias driven by unobserved covariates. We acknowledge that like most of the literature, we are unable to address leakage and spatial spillovers effects. Leakage would occur in our context if a forest or type of forest experienced increased degradation partly because another was better protected [26]. Spatial spillovers would occur if CFP management norms “spilled over” to non-CFP areas, affecting management and potentially outcomes. To our knowledge, no paper has formally addressed these issues of leakage and spatial spillovers in the community forestry context, though Bluffstone et al. [24] identified them as research priorities. We thank an anonymous reviewer for raising this issue.

## The Nepal community forestry program

Nepal covers a total of 147,148 square kilometers, approximately 40.4% of which is forested [11], and is divided into three geographic regions: the high Himalayas (16%) to the north, the middle hills (68%), and the lowland Terai (17%) to the south. Altitude ranges from 73 to 8,848 meters above mean sea level and includes diverse geo-climatic zones. While Nepal occupies 0.1% of the Earth’s land area, it harbors >3% of the world’s known flora and >1% of the fauna [27]. More than two-thirds of the population lives and works in rural areas within subsistence agricultural economies in which forests are important assets. Biomass energy produces approximately 70% of the total energy used, mainly from forests; 86% of households use fuelwood and 75% collect it themselves [28]. The burning of biomass fuels represents one of the major sources of greenhouse gas emissions.

After the nationalization of all forests in 1957, most Nepalese forests became open access, resulting in a version of the ‘tragedy of the commons’ [29]. Conservationists, scientists and administrators expressed alarm about the rapid deterioration of the Himalayan environment in the late 1960s and 1970s [30]. Gradually, local communities in the hills started protecting their forests to sustain flows of forest products [31], and the government and donors provided support. The Nepalese government began experimenting with early versions of the CFP in the mid-1970s and fully developed and implemented the program in the early 1990s. In the Forest Act of 1993, local forest-managing communities, called community forest user groups (CFUGs), are legally recognized as autonomous public bodies that can acquire, possess, transfer, and manage property [6]. Communities apply to become part of the CFP. If approved, five-year management plans are developed and implemented.

The Nepalese government prioritized certain forests for devolution under the CFP. For instance, the CFP was primarily promoted in the middle hill region, because many communities depended on forests and it was believed they were willing to protect them. There also existed traditional management practices, inability of government forestry staff to protect and manage forests, deteriorating forest conditions, little value for commercial uses, and financial and technical support from international organizations [31, 32].

In the lowland Terai, the government largely maintains control and has been reluctant to hand over forests to communities due to high commercial values and revenue potential [31, 33]; mainly small to medium sized forests in the vicinity of settlements were therefore handed over to communities [34]. The government particularly promoted the CFP in remote locations, for pine plantations [35] and in degraded areas [36]. Preference was also given to local communities that were cohesive, stable and traditionally managing forests [31].

As communities opted into the CFP and the government has demonstrated clear preferences for where forest devolution occurred, the assignment of communities to the CFP is not random. This important feature of the CFP implementation experience is addressed in our choice of analytical methods.

## Materials and methods

### Ethical considerations

As our research involves human participants, we carefully considered and addressed ethical issues and followed an institutional review board approval process at Portland State University. Because of the low literacy rate of Nepalese adult villagers (e.g., overall literacy rate in Nepal is 61% and the literacy among people older than 15 years, our potential subjects, is only 56%) [37] who are not accustomed to dealing with written forms, the institutional review board approved the use of verbal consent. Verbal consent was appropriate, as signed written consent forms are not consistent with local capabilities and traditions and generally make villagers uncomfortable.

Before seeking consent from potential subjects, we explained the study verbally, providing all pertinent information regarding research objectives, procedures, risks, benefits, and alternatives to participation, and allowed the potential subject ample opportunity to ask questions. When required or appropriate, we also provided a study information sheet and offered sufficient time to consider whether or not to participate in the research. Only if subjects voluntarily agreed did we conduct the survey.

As our field sites were in community-based forests, we sought permission from each community to go into their forests and conduct research. This approval came from village leaders or officers of CFUGs. Research did not involve endangered or protected species. Field research was implemented by ForestAction Nepal, a Nepalese action research organization.

### Sampling methods and research sites

By conducting a pilot survey in 45 forest plots from nine CFP forests across physiographic regions that captured the greatest possible variance in the basal area (a proxy for AGC), we estimated that 325 sample plots in CFP forests are required for our study to be representative at the national level. We calculated the number of required sample plots for 10% error and 95% confidence level using the standard formula given in Eq 1 [38].

$$N=C_{v}{}^{2}t^{2}/E^{2}$$ (1)

Where,

N = Required number of sample plots;

C_v_ = Coefficient of variation, s/μ (s = standard deviation and μ = sample mean);

E = Standard error, s/√n (n = sample number);

t = Value of student-t distribution for (n-1) degree of freedom and 95% confidence level.

Sample plots were distributed across 65 CFs, which were randomly selected from a larger random sample chosen for a national CF impact evaluation [39]. This CF impact evaluation was carried out by the Ministry of Forest and Soil Conservation of the Government of Nepal in collaboration with donors. The statistical bureau of Nepal selected a nationally representative, statistically valid random sample of 137 CFUGs across 47 of Nepal’s 75 districts. All sample CFUGs were established before 2005, and were selected from all ecological regions using stratified random sampling, with weights based on forest cover and CFUG size. Depending on forest size, we sampled between three and seven plots in each forest based on the quintile of the forest size distribution in which each forest resided. As forest size in the hills and Terai markedly differ, we used different quintile ranges (Table 1).

**Table 1:** Distribution of sample plots in community forests.

Quintile Distribution	Forest size (ha)		Sample Plots/Forest	Number of Forests	Number of Plots
	Hill	Terai			
1^st^ quintile	<18	<113	3	13	39
2^nd^ quintile	18–64	113–154	4	13	52
3^rd^ quintile	64–91	154–335	5	13	65
4^th^ quintile	91–183	335–526	6	13	78
5^th^ quintile	≥183	≥526	7	13	91

In addition to CFs, we selected 65 non-community forests (NCFs) and associated communities to be as similar to the CFs as possible based on ecological and social characteristics. NCFs were chosen to be close to, but not next to, CFs to avoid being used simultaneously by the same people. The resources of these NCFs are formally owned by the government, which also has management responsibilities. Local communities may protect and use forest resources, particularly non-timber forest products, but often NCFs are effectively open access.

**Fig 1:**
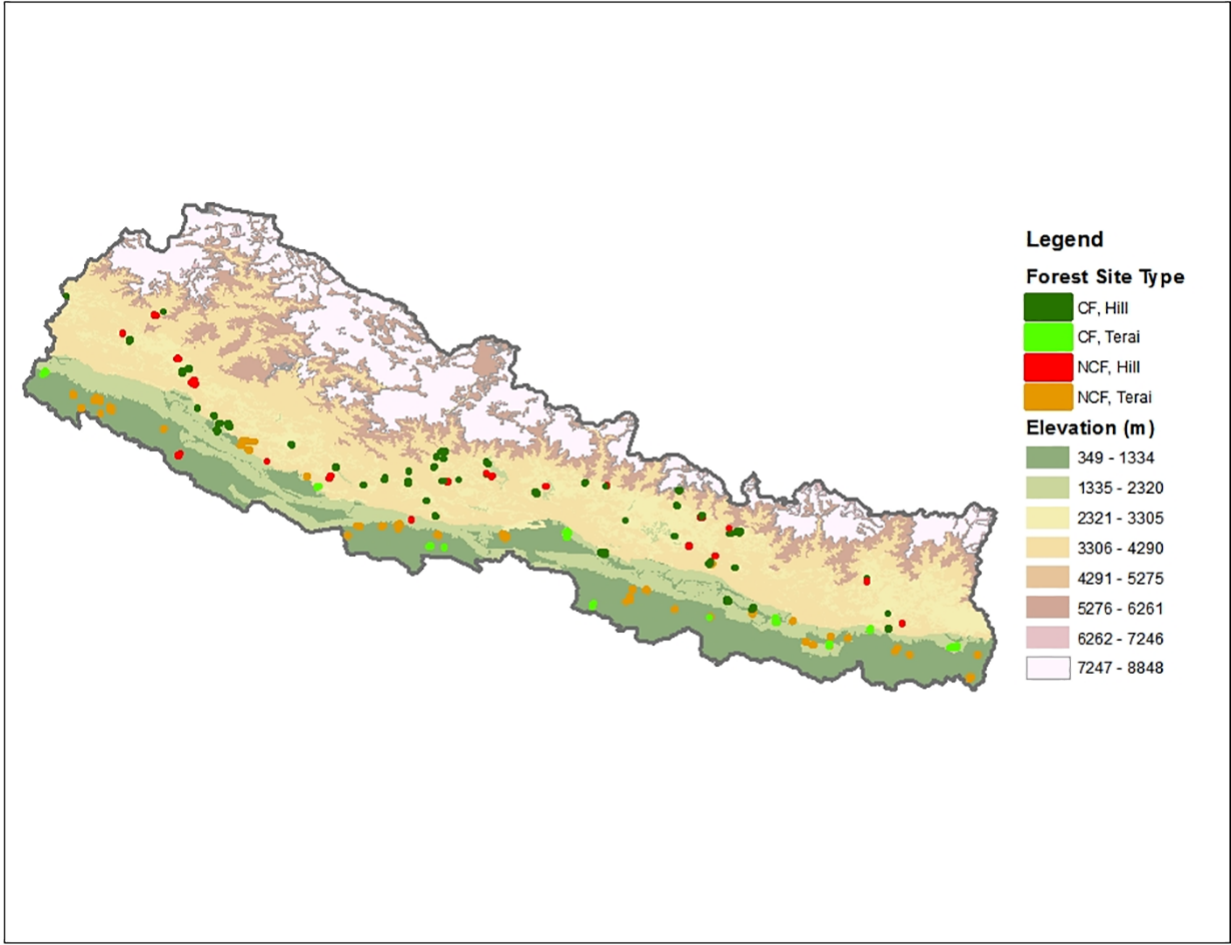
Distribution of sample plots across the country.

The field team surveyed the forest boundary of NCFs using Geographic Positioning Systems (GPS), prepared forest maps on graph paper, and estimated forest areas. The maps of CFs that were in the forest operational plan were also divided into smaller grid cells. To identify sample plots, cells from the maps were chosen randomly, and X and Y coordinates for the centers of selected cells were identified. The coordinates were then fed into a GPS unit to locate the plots in the forests. We randomly chose 295 NCF plots following the forest size criteria given in Table 1, as field logistical complexities precluded sampling 325 plots as was done for CFs. As suggested by Fig 1, the distribution of sample plots is representative of the Terai and middle hill regions of Nepal (Forest plot GPS coordinates are attached in S1 Table).

A circular plot with a radius of 8.92m was selected for collecting environmental data and measuring trees (>5cm diameter at breast height [DBH]), which is suitable for moderate to dense vegetation and is widely used [40]. Using the same center of the circular plot selected for collecting environmental data and tree measurements, concentric circles with radiuses of 5.64m and 1m were marked and used to measure saplings (1-5cm DBH) and count seedlings, respectively.

### Variables selection and measurement

Data collection was carried out from February to May 2013 by a team of 25 Nepalese field researchers, who either had undergraduate degrees in forestry or graduate degrees in social science. ForestAction Nepal recruited and trained them to conduct forest surveys, forest inventories, and social surveys. The team collected data about seedlings (species and count), saplings (species, count and diameter at breast height (DBH)), trees (species, count, dbh and height), and the environment (slope, altitude, aspect, canopy cover, soil erosion). The team also collected data regarding area and location of the forest, travel time required to reach the nearest road and district headquarters, population structure (poor, ethnic population, migration status), and community forest conservation duration (Questionnaire used to collect information is attached in S1 Text).

In addition, we used data from the community forestry impact study [39] and a cloud free Landsat 5 image from November 1989 to calculate normalized difference of vegetation index (1989 NDVI). NDVI is a widely used metric that estimates "greenness" of vegetative cover from remotely sensed data and is directly related to energy (visible light in the red band) absorption by plant canopies (chlorophyll) for use in photosynthesis, which correlates with denser vegetation [41, 42]. We chose 1989 NDVI, because this was before any of our communities had formed CFs and four years before the law creating the CFP was enacted; it therefore gives information about the quality of forests just before the CFP began. The measurement units and descriptive statistics of the data are presented in Table 2 and briefly discussed below. The community level data collection questionnaires from non-CFUGs (both in Nepali and English) and the forest data collection format are provided in the S1 Text.

**Table 2:** Descriptive statistics.

	Overall forest (N = 620)	CF (N = 325)	NCF (n = 295)
**Variables Measured at Plot Level**			
Altitude (m) directly measured using altimeters	748.20±25.20	981.67±33.77	485.13±31.69
Slope (degree) based on direct measurement	15.40±0.53	20.37±0.68	9.87±0.71
Moisture gradient (1–5 = low-high based on aspect, with south-facing having lowest moisture)	3.38±0.05	3.14±0.07	3.65±0.07
Presence of soil erosion (yes/no) as noted by field enumerators	158(25.5%)	78(24.0%)	80 (27.1%)
Average tree height (m) measured using clinometers	11.60±0.22	11.17±0.29	12.13±0.34
Average tree DBH (cm) measured using D-tapes	21.11±0.47	19.62±0.57	22.84±0.77
Canopy cover (%) subjectively assessed by trained enumerators	49.70±0.93	48.66±1.21	51.09±1.43
Tree density (no. ha-1)	570.11±18.14	629.17±27.75	503.46±22.40
Sapling density (no. ha-1)	491.73±22.04	512.92±35.67	471.19±25.07
Regeneration density (no. ha-1)	32316± 1369	29661± 1965	35420± 1896
Presence of *Shorea robusta* (yes/no)	350 (56.5%)	145 (44.6%)	205 (69.5%)
Broadleaved-conifer forest gradient (1 = broadleaved, 2 = mixed, 3 = conifer)	1.39±0.02	1.53±0.03	1.22±0.03
Normalized Difference Vegetation Index-NDVI, 1989 (0 = bare, 1 = green) computed from 1989 LANDSAT imagery	0.2942±0.0022	0.2945±0.0030	0.2938± 0.0032
**Variables Measured at Forest or CFUG Level**			
Forests are in the hills (yes/no) from documents	264 (42.58%)	205 (63.08%)	59 (20.00%)
Forests are in Terai (yes/no) from documents	356 (57.5%)	120 (36.92%)	236 (80.00%)
Years of forest user group formation (no.) from survey	11.2±0.20	10.10±0.26	12.43±0.28
Number of forest users households from document	295.82±101.09	295.80±182.70	295.85±88.44
Forest area (ha) from CFUG documents (for CF) or directly measured using GPS measurements (for NCF)	127.70±27.92	148.96±44.17	106.44±33.71
**Variables Measured at Household Level and Aggregated to the Forest or CFUG Level**			
Time required for 2-way travel from road to forest (1 = < 2 hours, 2 = 2 hours to half day; 3 = > half day)	1.41±0.03	1.48±0.05	1.33±0.04
Time required for 2-way travel to and from district headquarters to forest (1 = < 2 hours, 2 = 2 hours to half day; 3 = > half day) (reported by respondents)	2.45±0.05	2.59±0.07	2.28±0.06
Households living in the village for at least 2 generations (proportion	0.748±0.011	0.821±0.014	0.668±0.017
Indigenous/ethnic population (proportion)	0.416±0.012	0.437±0.019	0.394±0.015
Poor population (proportion)	0.376±0.009	0.372±0.012	0.382±0.014
**Variables Calculated Based on Field Data**			
Per household forest area (ha) from CFUG documents (for CF) or directly measured using GPS measurements and communities’ records (for NCF)	0.82±0.30	0.90±0.27	0. 47±0.32

Continuous and ordinal variables are presented as means ± standard errors of the mean; dichotomous variables are presented as n (%).

Sample plots were distributed in the tropical, sub-tropical and temperate climatic zones of 42 districts (out of 75) and 130 forests across the country. Though the majority of sampled forests are in the Terai (57.5%), a majority of CFs (63%) are in the hills, reflecting the higher number and area of CFs in the hills, i.e. ~87% of CFs covering and ~80% CF area are in the hills [43]. The sample plots ranged from approximately 80m to 2800m altitudes (average 748.20±25.20m) above mean sea level and 0 to 60-degree slope (average 15.40±0.53 degree). While the average size of forests in the overall sample is 127.70±27.92 ha, the average number of households per forest is 295.82±101.09 (average forest size per household = 0.82±0.30 ha). The mean time required to travel to and from forests to the nearest road-head is less than half day, while the time required to travel to the district-headquarters is more than half day. The average moisture gradient, reflected primarily by aspect, is modest.

We group our variables into three main categories–treatment/control, outcomes and confounding.

#### Treatment and control variables

The implementation of a formal CFP (or CF) is the treatment and non-implementation of the CFP (or NCF) is the control variable. Though the CFP has been implemented in Nepal since the mid-1970s in different models, the current model has been implemented since the early 1990s and was written into law in 1993.

#### Outcome variables

The effective number of species (e^H’^) and the Aboveground Tree and Sapling Carbon (AGC) are the two outcome variables. We used e^H’^ (i.e. numbers of species present if all species were equal in abundance) to assess biodiversity as it is an unbiased estimate of biodiversity that reduces inaccuracies when comparing diversity across plots [44]. It measures biodiversity, considering both species richness (S) and abundance, in units of the number of species, making it relatively easy to interpret.

We checked the names of all tree and shrub species for orthography and synonymy. We calculated S by simply counting the number of species present in a plot. To estimate e^H’^, we calculated the Shannon-Weiner Index (*H’)* using Eq (2). H’ is positively correlated with the number and evenness of species and takes a value of zero when there is only one species and a maximum value when all species are present in equal abundance.

$$H’=-\Sigma^{S}{}_{i=1}\;p_{i}\;\ln\;p_{i}$$ (2)

Where, *S =* species richness;

*i =* individual species;

*p*_*i*_ *=* individuals of one species (n) divided by the total number of individuals of all species in the plot (N);

Σ = sum of the calculations.

AGC provides information about the location of carbon sources and sinks, particularly providing an estimate of major carbon storage in forests and potential emissions from deforestation and forest degradation [45, 46]. Recent studies using estimates of AGC indicate a large potential for tropical forests to serve as carbon sinks [47, 5].

We use Eqs (3) and (4) to estimate Aboveground Biomass (AGB). These equations were prepared using a large global dataset of trees across different climatic conditions, to estimate AGB in dry (<1500mm average annual rainfall) forests (Eq 3.) and moist (1500-4000mm average annual rainfall) forests (Eq 4) [48]. These equations are recommended by the Nepalese government [49]. Approximately 5% of sample plots are in dry forests.

$$AGB\;\left(kg\right)=0.112\;*\;{\left(\rho D^{2}H\right)}^{0.916}$$ (3)

$$AGB\;\left(kg\right)=0.0509\;*\;\rho D^{2}H$$ (4)

Where,

ρ = Specific gravity of wood (g cm-3);

D = DBH;

H = Tree height.

We used species-based wood specific gravity to calculate biomass [50]. Where such information was unavailable, we used values derived from average specific gravity of associated species (same genus and family) within a forest type [51, 52, 53]. We used Nepal-specific biomass equations to estimate the green biomass of individual species [54]. We converted the green biomass into dry biomass by multiplying by species-specific fractions or the average of associated species identified in the literature. We used the fractions 0.627, 0.613, 0.58, 0.57, 0.545, 0.517, 0.5 and 0.45 for *Quercus* species, *Lyonia ovalifolia*, *Pinus roxburghii*, *Alnus nepalensis*, *Schima wallichii*, *Shorea robusta*, *Terminalia tomentosa* and *Pinus wallichiana*, respectively [55, 56, 57, 58, 59]. For unidentified species, or where wood density information was not available for the species, genus or family, we used the overall mean wood density obtained from the database [51] for the species identified by our field team. Finally, we converted AGB into carbon by multiplying by 0.50 [60].

#### Confounding variables

Because communities chose to participate in the CFP, there are a number of potential confounders that may affect treatment status and/or outcomes. On the basis of the literature, focus group discussions with 20 forest-managing communities and one consultation meeting in Kathmandu in 2012, we identified 16 observable confounders determined by forest/topographical features and community characteristics (Table 2). These variables include the most important biophysical information (e.g. average slope, 1989 NDVI, forest area, average moisture gradient, tree species, including the presence of commercially important *Shorea robusta*, and altitude) affecting CFP assignment.

The government also had some key socioeconomic choice criteria (e.g. remoteness, experience with conservation, stability of communities) when granting CFP status. These variables are therefore included in our matching models and are listed in Table 2.

It was impossible to capture all features that could determine CFP assignment; unobservable variables (e.g. local capacity, willingness to protect forests and/or the presence of capable leaders) affected whether forests and communities were part of the CFP. We therefore conduct sensitivity analysis to evaluate the degree to which a key model assumption, that CFP assignment is random after matching, must be violated in order for results to be reversed. This analysis provided evidence regarding the degree to which we have included the essential drivers of CFP assignment in our matching models.

Because we collected forest, topography, and community data at forest, forest plot, and community scales, we used mixed effect probit models to examine the relationship between these confounders and CFP assignment for the overall sample and forest subsamples defined by altitude (higher and lower), slope (higher and lower), geo-political region (hill and Terai) and canopy (open and closed). We characterize the forest plot by altitude, slope, geo-political region, and canopy cover and results are presented for subsamples as well as the overall sample. Plot altitudes ranged from 75m to 2775m above mean sea level. Based on the change in vegetation by altitude [12], we disaggregated altitudes into lower altitude (<1000m) and higher altitude (**≥**1000m). Slope ranged from 0 to 60 degrees (very few over 40^**0**^). We therefore categorized forests into lower slope (< 15^**0**^) and higher slope (**≥** 15^**0**^) to account for slope-related differences in forest type, structure and composition. We also disaggregate results according to Terai and hill using the official government categorization. Following general practice, we categorize plots into open canopy (< 50%) and closed canopy forests (**≥**50%). Mixed effect probit is appropriate, because our unit of analysis is the plot level, but the level of CFP assignment is the forest level. Model results for the log odds ratio of CFP assignment are presented in Table 3.

**Table 3:** Potential confounders and their relationships with CFP assignment. CFP Status is the Dependent Variable.

Possible Confounder	Overall forest (n = 620)	Lower altitude <1000m (n = 413)	Higher altitude ≥1000m (n = 207)	Low slope (<15^0^) (n = 277)	High slope (≥ 15^0^) (n = 343)	Terai (n = 356)	Hill (n = 264)	Open canopy (<50%) (n = 276)	Closed canopy (≥ 50%) (n = 344)
Intercept	-6.07 (10.40)	-15.83 (10.47)	-4.90 (21.82)	0.65 (14.06)	-17.04 (17.04)	-26.95 (20.08)	**-32.64** (0.002)	7.95 (8.93)	-9.05 (13.94)
Forest area	0.02 (0.01)	0.02 (0.01)	0.04 (0.07)	0.01 (0.01)	0.07 (0.04)	0.01 (0.01)	**0.28** (0.001)	0.01 ((0.01)	**0.03** (0.01)
Forest size per household	1.41 (1.04)	1.60 (1.25)	3.28 (7.53)	2.49 (1.38)	0.17 (1.78)	2.24 (1.46)	**-5.87** (0.002)	**3.34** (1.39)	1.15 (1.01)
Travel time to nearest road	-1.43 (2.00)	-3.55 (2.93)	0.04 (2.63)	1.85 (2.90)	-4.02 (2.35)	5.85 (3.63)	**-3.54** (0.002)	-0.41 (2.37)	-1.54 (2.27)
Travel time to district headquarters	1.23 (1.15)	0.78 (1.34)	-1.46 (2.41)	-1.85 (1.45)	2.33 (1.45)	**-5.93** (2.22)	**3.39** (0.002)	0.75 (1.54)	1.39 (1.00)
Slope	0.08 (0.07)	0.09 (0.09)	0.02 (0.12)	0.52 (0.37)	0.09 (0.09)	0.06 (0.10)	**3.11** (0.002)	0.07 (0.08)	0.14 (0.08)
Altitude	**0.01** (0.002)	**0.02** (0.01)	-0.0003 (0.004)	**0.01** (0.004)	**0.01** (0.004)	**0.014** (0.005)	**0.004** (0.001)	0.01 (0.003)	0.01 (0.004)
Moisture gradient	-0.17 (0.42)	-0.32 (0.55)	0.63 (0.89)	-0.75 (0.95)	0.17 (0.50)	-0.24 (0.65)	**0.44** (0.002)	0.16 (0.54)	-0.44 (0.48)
Broadleaved-conifer gradient	0.49 (2.60)	**9.04** (4.190	-6.93 (5.24)	1**1.19** (4.76)	-1.48 (2.41)	**11.17** (4.85)	**-4.55** (0.002)	1.67 (3.03)	0.21 (3.55)
Presence of *Shorea robusta*	-1.07 (1.74)	-1.75 (2.50)	-1.30 (3.710	-6.66 (5.10)	-0.21 (2.24)	-1.73 (2.65)	**-2.80** (0.002)	-0.09 (1.84)	-0.67 (2.66)
Presence of soil erosion	-1.24 (1.52)	-0.08 (2.04)	-0.28 (2.08)	-0.24 (3.17)	-1.17 (1.83)	-0.95 (2.34)	**-3.03** (0.002)	0.46 (1.94)	-1.14 (1.60)
1989 NDVI	-14.29 (15.61)	-14.45 (14.71)	6.09 (31.54)	-15.70 (17.36)	1.05 (20.02)	16.94 (28.12)	**3.58** (0.002)	-24.91 (14.98)	-2.29 (22.62)
Communities conservation duration	**-0.75** (0.22)	-0.16 (0.41)	**-0.81** (0.37)	**-1.00** (0.50)	**-0.74** (0.27)	-0.73 (0.45)	**-1.10** (0.002)	**-1.12** (0.34)	**-0.58** (0.24)
Forest user households	**0.003** (0.001)	**0.003** (0.001)	0.02 (0.02)	**0.003** (0.001)	0.004 (0.02)	0.003 (0.002)	0.02 (0.002)	**0.003** (0.001)	**0.003** (0.001)
% of households living in the village for ≥2 generations	10.64 (6.35)	0.68 (5.10)	27.35 (20.31)	0.990(5.25)	10.00 (9.73)	6.20 (6.18)	**36.10** (0.002)	7.04 (5.44)	6.29 (5.99)
% of indigenous population	1.59 (4.28)	9.25 (6.72)	0.07 (4.44)	9.07 (6.10)	0.80 (4.35)	**15.74** (7.37)	**2.94** (0.002)	3.59 (4.55)	-2.63 (4.15)
% poor population	-1.340 (6.12)	-3.71 (6.48)	-5.46 (11.19)	-16.71 (8.87)	4.72 (6.75)	-15.51 (10.38)	**6.92** (0.002)	**-17.46** (7.00)	-0.72 (6.42)

Standard errors are in parentheses and statistically significant estimates (p-values ≤ 0.05) are in bold.

All 16 variables related to forest area and location, species composition, 1989 NDVI, number of households in the community, communities’ conservation duration, and population structures have coefficients that are significantly different from zero in at least one of the models, suggesting that CFP assignment evaluated at the forest plot level was largely non-random (Table 3). For instance, the log odds ratios of a forest being selected into the CFP increased by 0.28 for each additional hectare of forest in the hill and 0.03 in closed canopy forests. This may reflect local communities’ preference for larger forests, and the government’s policy of handing over forests to local communities according to communities’ willingness to manage [61].

Similarly, the log odds ratio of travel time from the forest to the nearest road (lower slope, higher altitude, and Terai forests) and district headquarter (overall, hill, lower altitude, high slope, open canopy, and closed canopy forests) for CFP assignment are positive, reflecting higher probabilities of remote forests being assigned to the CFP [31]. The positive, significant log odds ratio of broadleaved-conifer gradient (overall, lower altitude, lower slope, Terai, open canopy, and closed canopy forests), reflects the higher probability of pine forests being handed over to communities [35]. In addition, we estimated negative log odds ratios for forests being selected as CFs if the number of years communities conserved forests increased; and positive log odds ratios for forests being selected as CFs if the number of forest users’ households or the proportion of ancestral population or the proportion of indigenous/ethnic population increased.

In contrast, the log odds ratio for CF selection declines as NDVI increases (overall, lower altitude, lower slope, open canopy, and closed canopy forests). This confirms that degraded forests were more often handed over to communities [36]. Similarly, the log odds ratio declines as the proportion of poor population increases in the community except in the hills and high slopes.

If the treatments were randomly assigned, estimated log odds ratio would be statistically insignificant or zero for these confounding variables. However, these confounders were an important decision criteria during the initial years of the CFP and were found to be statistically significant for one or more of the forest categories. We therefore include them in our analytical models unless they had to be dropped to achieve balance.

### Addressing confounding through matching

Because communities chose to participate in the CFP, there were a number of potential confounders that may affect treatment status and cause bias in ATT_B_ and ATT_C_ estimates [62]. Controlling the effects of confounders through the matching process, an ex post identification technique, is critical to minimize bias and identify the best CF and NCF plot comparators. Use of data from NCF plots to develop a counterfactual control group to estimate what would have happened to biodiversity and carbon storage in the absence of CFP is critical [63].

We used a nonparametric matching method, which reduces selection bias and generates a comparable set of NCF observations by controlling 10–14 observed confounders in different categories of forests [64, 65, 66, 67]. We used MatchIt package of R 3.2.2 (Ho et al., 2007), where we input mixed-effect probit outcomes and utilized matching with replacement that produces the highest degree of balance and lowest conditional bias [68, 69].

We also used a “genetic matching” algorithm that optimizes matches by automating the process of finding good matches using an evolutionary search algorithm [70]. Genetic matching generalizes the propensity score and Mahalanobis distance matching [71] and maximizes balance using p-values even when there are several, correlated confounders [71, 72]. We used standardized mean difference (SMD), which expresses the standardized bias and is similar to an effect size relative to the variability observed, for finding acceptable matches of CF and NCF plots based on observed confounders. SMD was estimated by dividing the difference in mean outcomes between CF and NCF plots by standard deviations of outcomes across CF plots. A smaller SMD minimizes overt bias in ATT_B_ and ATT_C_ estimates due to measured covariates [73, 74] and therefore we used ≤0.25 SDM as a cut-off point for matching adjustment, a common numerical balance diagnostic criterion to check whether matching is satisfactory and acceptable [75]. We found a sufficient number of NCF plots (18–52% in different categories of forests) that are similar to CF plots. Post-matching average SMDs for different confounders ranged from 0.08–0.17 (S2 Table).

### Estimating effects on biodiversity and carbon, and sensitivity of results

Because the difference in e^H’^ and AGC between matched CFP and non-CFP plots are not normally distributed, we used a pairwise Wilcoxon signed rank sum test to estimate median ATT_B_ and ATT_C_. We also estimated how robust our ATT_B_ and ATT_C_ estimates are in view of potential unobserved confounders, following the sensitivity analysis approach from the sensitivitymv package in R 1.3 [76, 77]. Using this method, we quantified the degree to which a key model assumption—that CFP assignment is effectively random conditional on the matches—must be violated in order for results to be reversed.

We used a sensitivity parameter, gamma (Γ), that shows critical levels of hidden bias as a measure of difference in the odds ratio of CFP assignment for two plots with the same observed confounders, but that diverge on unobserved confounders. We then determined the smallest value of Γ that would change the p-value of the “true” ATT_B_ or ATT_C_ to a non-significant level (>0.05); the Γ value at this point indicates the CF to NCF odds ratio at which ATT_B_ or ATT_C_ estimates are sensitive to hidden bias. A higher Γ implies that the estimated ATT_B_ and ATT_C_ results are more robust to potential hidden bias, while a low Γ suggest that even a mild hidden bias could make the estimate insignificant and Γ = 1 indicates even no hidden bias or that full randomization could overturn results.

## Results

Biodiversity in CFP plots was significantly (p<0.05) higher than non-CFP plots in the overall forest, lower and higher slopes, open canopies and Terai (Table 4). Positive ATT_B_ indicates significant positive effects of the CFP on e^H’^, with point estimates ranging from 0.60–0.88 in lower slopes, overall forests, higher slopes, Terai and open canopies (Table 4). We found no significant effect of the CFP on biodiversity in lower altitudes, hills and closed canopies. The overlapping estimates of ATT_B_ within the 95% confidence interval (CI) indicate that there is no difference in ATT_B_ when comparing overall forest, lower altitudes, higher slopes, open canopies and Terai subsamples (Table 4).

**Table 4:** Average effect of CFP on e^H’^ and sensitivity analysis by forest category.

(1) Forest Category	(2) No. of Plots in CFP/ non-CFP	(3) Average SMD of Observed confounders (before/ after match)	ATT_B_ (Comparison of Medians)				Hidden bias
			(4) Point estimate	(5) Lower confidence limit-95%	(6) Upper confidence limit-95%	(7) p-value	(8) Critical level of bias (Γ)
Overall forest	325/70	0.40/0.11	0.65	0.31	1.00	**0.000**	1.24
Lower altitude	170/60	0.37/0.21	0.38	-0. 14	0.90	0.151	
Higher altitude	155/28	0.24/0.08	-0.51	-0.98	-0.04	**0.031**	1
Lower slope	89/28	0.39/0.11	0.60	0.08	1.14	**0.024**	1.18
Higher slope	236/56	0.26/0.17	0.67	0.27	1.07	**0.001**	1.36
Terai	120/43	0.36/0.13	0.73	0.20	1.22	**0.008**	1.26
Hill	205/41	0.16/0.10	-0.29	-0.71	0.17	0.201	
Open canopy	149/41	0.42/0.09	0.88	0.39	1.36	**0.001**	1.45
Closed canopy	176/53	0.39/0.13	0.33	-0.04	0.07	0.072	

Columns 2 and 3 provide the number of CFP/non-CFP forest plots and average SMD of confounders before and after matches. Columns 4, 5, 6 and 7 present the ATT_B_, lower and upper confidence levels of ATT_B_ and p-values. Columns 8 provide information about the sensitivities of estimated results to unobserved confounders and the p-values.

For sensitivity estimation, trimming was carried out at 2.5 times the median of the absolute matched difference, which is analogous to a trimmed mean that trims 5% of outliers from each tail. As there was no need, we did not calculate the hidden bias for insignificant CFP effects.

Sensitivity analyses indicate that unobserved confounders may nullify these results if the odds ratios of CFP to non-NCP forest plots are changed to 1.24, 1.18, 1.36, 1.45 and 1.26 in overall forest, and forests in lower slopes, higher slopes, open canopies and Terai, respectively (Table 4). These odds ratios suggest that our estimates are quite robust to hidden bias or effects of unobservable confounding variables. Open canopies had the lowest average standard mean difference of confounders (SMD) (0.09), narrower CI of ATT_B_ (0.02–0.24) and higher CFP to non-CFP odds ratios (1.45) (Table 4), indicating that the ATT_B_ estimate was improved by better matched CF and NCF plots, and was more precise and less sensitive to hidden bias than forests in other categories. Such statistics suggest a highly robust ATT_B_ estimate in open canopies.

For lower altitudes, closed canopies and hills, our estimates indicate that ATT_B_ in CFP and non-CFP plots are not significantly different at the 5% level. Biodiversity in CFP and non-CFP forest plots with higher slopes were statistically different, but we found a negative (-0.51) effect of CFP status on e^H’^. Sensitivity analysis shows, however, that this result can be nullified by the influence of unobserved confounders even if forest plots were fully randomized, which suggests that our estimate of ATT_B_ for higher slopes is not robust to unobservable confounders.

### Effect of the CFP on carbon

In contrast with ATT_B_ estimates, which are relatively similar across forest categories, our ATT_C_ and levels of sensitivity vary across forest categories. There are significant negative effects of the CFP on AGC at the national level (-15.11 Mg C ha^-1^) and for higher slopes (-17.72 Mg C ha^-1^). However, unobserved covariates may nullify these results even if plots in both CFP and non-CFP forests were fully randomized. In lower altitudes, Terai, hills and closed canopies, CF and NCF plots had similar levels of ATT_C_ (Table 5).

**Table 5:** Average effect of the CFP on AGC and sensitivity analysis by forest category.

(1) Forest Category	(2) No. of plots in CFP/ non-CFP	(3) Average SMD of Observed confounders (before/after match)	ATT_C_ (Comparisons of Median)				Hidden bias
			(4) Point estimate	(5) Lower confidence limit-95%	(6) Upper confidence limit-95%	(7) p-value	(8) Critical level of bias (Γ)
Overall forest	325/70	0.40/0.11	-15.11	-26.35	-3.49	**0.012**	1
Lower altitude	170/60	0.37/0.21	11.21	-7.42	31.02	0.243	
Higher altitude	155/28	0.24/0.08	-22.81	-37.41	-9.39	**0.001**	1
Lower slope	89/28	0.39/0.11	25.51	0.98	55.14	**0.041**	1.10
Higher slope	236/56	0.26/0.17	-17.72	-30.93	-4.22	**0.010**	1
Terai	120/43	0.36/0.13	5.87	-15.88	32.80	0.585	
Hill	205/41	0.16/0.10	9.76	-1.48	22.04	0.089	
Open canopy	149/41	0.42/0.09	25.84	12.22	41.36	**0.000**	1.66
Closed canopy	176/53	0.39/0.13	-2.93	-18.06	12.11	0.694	

Columns 2 and 3 provide the number of CFP/non-CFP forest plots and average SMD of confounders before and after matches. Columns 4, 5, 6 and 7 present the ATT_C_, lower and upper confidence levels of ATT_C_ and p-values. Column 8 provides information about the sensitivities of estimated results to unobserved confounders and the p-values.

ATT_C_ was significantly (p<0.05) higher in CFP than non-CFP plots in lower slopes (25.51 Mg C ha^-1^) and open canopies (25.84 Mg C ha^-1^). The sensitivity analysis indicates that unobserved confounders may nullify these results if the odds ratios of CFP to non-CFP plots were changed to 1.10 and 1.66 in lower slopes and open canopies, respectively (Table 5).

The 95% confidence interval comparing forest categories indicates that ATT_C_ in the overall forest is less than in lower slopes, open canopies and hills, but is not statistically different from other categories of forests (e.g. lower altitudes, higher altitudes, higher slopes, Terai and closed canopies). ATT_C_ in lower slopes and open canopies was not statistically different. However, open canopies had the lowest estimated SMD (0.09), narrower CI of ATT_C_ (12.22–41.36) and, a higher CF to NCF odds ratio (1.66) (Table 5), indicating that the ATT_C_ estimate was improved through better matched CF and NCF plots, was more precise, and was less sensitive to hidden bias than forests in lower sloped areas. Such statistics suggest a highly robust ATT_C_ estimate in open canopies.

## Discussion

At the national level, our results suggest that the CFP has a positive effect on e^H’^ and, though not robust to unobservable factors, has a negative effect on AGC. However, the CFP has mixed effects on e^H’^ and AGC when disaggregated by altitude, slope, geographic region and canopy cover. We found marginal effect of CFP on e^H’^ (0.65) of 20.57% of the median e^H’^ of our overall sample (3.16). The generally positive effect of the CFP on biodiversity likely reflects the contribution of the CFP to revitalizing degraded forestlands [8, 10], but also depends heavily on communities’ efforts [17], reflecting the net effect of a variety of forest management actions carried out by communities. We found that the CFP affects forest management decisions such that across our full sample, biodiversity increased. We did not find evidence that the CFP pushed communities toward a limited selection of commercial species. Indeed, at the macro level, the opposite appears to be the case.

We found a marginal effect of CFP on AGC (-15.11 Mg C ha^-1^) of 23.43% of the median AGC of our overall sample (64.48 Mg C ha^-1^). Our estimates of the CFP’s effect on AGC should be viewed in the context of objectives and management practices in CFs, possible disturbance regimes, base carbon stocks, and potential spillover effects of CFs on NCFs, some of which might not have been well captured by our observables. After implementation of the CFP, communities primarily managed forests ‘passively,’ for forest products [19]. Such a management approach, while likely enhancing biodiversity, can limit carbon storage.

As extractions of timber and other wood products from CFP forests are legal, the carbon stored in CFs is what remains after harvesting. In contrast, harvesting timber and woody products in non-CFP forests is prohibited. Reduced AGC storage in CFs may therefore simply reflect communities exercising more secure extraction rights. This line of argument, however, has limited support, because many CFP forests, particularly in the hills, are passively managed and more biodiversity is unlikely to be consistent with increased timber extractions. Many non-CFP forests are *de facto* open access, which should cause more extractions [78, 29]. We therefore find it unlikely that relatively unregulated non-CFP forests are harvested less than CFP Forests.

There are at least two other reasons why CFP forests could have at best no more carbon than non-CFP forests despite more secure rights. First, we adjusted for base ecological status using the 1989 NDVI (a proxy for growth rates or carbon sequestration but not carbon storage) and found that CFP forests have statistically similar growth as non-CFP forests, but after adjusting for other factors (in some forest categories though not overall), CFP forests had statistically lower growth rates. If CFP forests initially had less carbon than non-CFP forests and NDVI does not provide an appropriate proxy for 1989 carbon storage, we omitted a variable that could bias downward our estimates of the effect of the CFP on carbon.

Second, NCFs in the vicinity of communities could have mimicked CFP forests with an aim to improve their forest management without participating in the CFP. They might also want to demonstrate their commitment to forest management as a way to persuade forestry officials to designate the forests they use as part of the CFP. This would be an example of a spatial behavioral spillover from CFs to NCFs. We have some evidence that this may be occurring. For instance, community and household surveys carried out as part of this research suggest that 80% of communities have written rules (which is required for CFP but not for non-CFP forests) and >60% of households in NCF communities engage in forest management.

With the exceptions of the high-altitude subsample, for which we estimated a negative ATT_B_ (though with Γ = 1 indicating that results are not at all robust to unobservable variables) and the hills, where we estimated no effect, in the full sample and subsamples we found positive and statistically significant ATT_B_. These are reasonably robust results, which suggest that the CFP likely increases plant biodiversity.

Our results were much less clear for carbon and only the open canopy subsample results are robust to unobservable variables. The potential for positive, negative or no contributions of the CFP to carbon stocks suggests the need for further assessment of CFPs and highlights the limitations of evaluating the CFP exclusively at a national scale. For example, the negative and significant ATT_C_ in the full sample was driven wholly by higher altitude and steeper-sloped plots, but AGC is higher or the same in lower and flatter plots. We believe this difference reflects the on-the-ground reality that in the middle hills, where steeper slopes and higher altitudes are found, CFs are ubiquitous and generally older than in the lowland Terai. Management plans may therefore differ, and it is possible that some steeper-sloped and higher altitude forests have negotiated management plans that allow for more biomass removal, while in the Terai, where there are higher value forests and generally newer CFs, CF harvests are more restricted.

Different results across geographic regions suggest that rather than a “one size fits all approach” to forest management, adaptive and area-specific policies and programs are critical for sequestering carbon and perhaps to a lesser extent, promoting biodiversity. Such policies and programs need to provide communities with practical guidance for adopting locally suitable management options. Our findings therefore challenge the current homogenous policy, which is in place despite potentially important geographic, topographic and forest quality differences. For instance, the same CFP rules are applicable for natural and plantation forests, large and small-sized forests, forests in high-hill, mid-hill and Terai and open-canopy and closed canopy forests. Our findings of different effects of the CFP across these settings suggest that policies and programs need to be tailored to local circumstances.

Estimating the ATT_B_ and ATT_C_ is challenging with the inevitable observational data, although matching based on a large number of potential confounders and sensitivity analysis increase our confidence in the results. Some limited imbalance in the observed confounders remained despite our best efforts, although SMD was always brought below generally accepted cut-off points. As there are several matching algorithms, each with strengths and weaknesses, there is always room for questions related to the quality of the matching. The use of SMD as a criterion to check the acceptability of the match balance could also be critiqued.

## Conclusion

We measured the causal effects of Nepal’s CFP on biodiversity conservation and carbon storage by employing a quasi-experimental matching method that reduces confounding bias and strengthens results. We found that Nepal’s CFP at the national level is associated with higher biodiversity, but perhaps lower carbon stocks. We also found heterogeneous (positive, negative or zero) effects of the program, particularly on carbon and to a lesser degree on biodiversity depending on region, altitude, slope and canopy cover. Such results suggest that the Nepalese CFP does not clearly offer a universal path to biodiversity conservation or carbon sequestration. Dedicated policies are crucial to incentivize communities to pursue active forest management that enhance biodiversity conservation and carbon storage.

This research broadens and deepens scientific understanding regarding the effects of forest decentralization on biodiversity conservation and carbon storage. It provides critical information to policy makers and managers for designing targeted, appropriate plans across regions to improve CFP outcomes in terms of biodiversity conservation and carbon storage. It also points to the need for future research that helps explore why forest decentralization is more effective in some areas than in others, how forest-managing communities interpret and apply forest decentralization policies, and what motivational and capacity building support is needed to make such policies compatible with contemporary global environmental initiatives, including the CBD and REDD+.

## Supporting information

S1 Table Forest plot GPS coordinates. (DOCX)

S2 Table Standardized mean difference before and after matching by covariate. (DOCX)

S1 Text Questionnaire used to collect information. (PDF)
