# Supplementary material for: The effects of the Nepal community forestry program on biodiversity conservation and carbon storage
Source: PLoS One. 2018 Jun 26;13(6):e0199526. doi: 10.1371/journal.pone.0199526 (PMC6019746; doi:10.1371/journal.pone.0199526)
Supplement: S1 Table — (DOCX) [file pone.0199526.s001.docx]

S1 Table. GPS coordinates of forest plots

| **District Name** | **Plot_number** | **Plot ID** | **CFUG_name** | **GPS X coordinate** | **GPS Y coordinate** | **Altitude**  **(meter)** |
| --- | --- | --- | --- | --- | --- | --- |
| Accham | 1 | 1 | Kalika | 536486 | 3209639 | 1422 |
| Accham | 2 | 2 | Kalika | 536886 | 3210089 | 1433 |
| Accham | 3 | 3 | Kalika | 536686 | 3210189 | 1500 |
| Accham | 4 | 4 | Kalika | 537186 | 3210389 | 1449 |
| Accham | 5 | 5 | Kalika | 536886 | 3210489 | 1487 |
| Accham | 6 | 6 | Kalika | 536686 | 3210689 | 1480 |
| Accham | 1 | 7 | Kokila | 532770 | 3222589 | 1675 |
| Accham | 2 | 8 | Kokila | 533270 | 3222689 | 1800 |
| Accham | 3 | 9 | Kokila | 533570 | 3222589 | 1505 |
| Accham | 4 | 10 | Kokila | 533470 | 3222989 | 1845 |
| Accham | 1 | 11 | Raniban | 542272 | 3213799 | 747 |
| Accham | 2 | 12 | Raniban | 542172 | 3213599 | 735 |
| Accham | 3 | 13 | Raniban | 541872 | 3213499 | 777 |
| Accham | 4 | 14 | Raniban | 541972 | 3213099 | 770 |
| Baglung | 1 | 15 | Dudewa Chaur | 723341 | 3120483 | 780 |
| Baglung | 2 | 16 | Dudewa Chaur | 723441 | 3120533 | 755 |
| Baglung | 3 | 17 | Dudewa Chaur | 723541 | 3120583 | 790 |
| Baglung | 1 | 18 | Jallibir | 755509 | 3111104 | 655 |
| Baglung | 2 | 19 | Jallibir | 755459 | 3111354 | 639 |
| Baglung | 3 | 20 | Jallibir | 755459 | 3111454 | 690 |
| Baglung | 1 | 21 | Jauchhare | 743719 | 3125506 | 2089 |
| Baglung | 2 | 22 | Jauchhare | 744319 | 3125306 | 1961 |
| Baglung | 3 | 23 | Jauchhare | 744119 | 3125206 | 2068 |
| Baglung | 4 | 24 | Jauchhare | 744219 | 3124906 | 2123 |
| Baglung | 1 | 25 | Majhkatera | 745735 | 3134496 | 2070 |
| Baglung | 2 | 26 | Majhkatera | 745935 | 3134446 | 2111 |
| Baglung | 3 | 27 | Majhkatera | 746085 | 3134146 | 2067 |
| Baglung | 4 | 28 | Majhkatera | 746285 | 3134746 | 2507 |
| Baglung | 3 | 29 | Mauribhir | 751716 | 3133191 | 1725 |
| Baglung | 4 | 30 | Mauribhir | 751623 | 3133276 | 1742 |
| Baglung | 5 | 31 | Mauribhir | 752107 | 3133135 | 1743 |
| Baglung | 1 | 32 | Nauley | 751833 | 3111547 | 1058 |
| Baglung | 2 | 33 | Nauley | 751732 | 3111647 | 1066 |
| Baglung | 3 | 34 | Nauley | 751233 | 3111847 | 1137 |
| Baglung | 4 | 35 | Nauley | 751433 | 3111747 | 1124 |
| Baglung | 5 | 36 | Nauley | 751133 | 3111747 | 1085 |
| Bajhang | 2 | 37 | Chiuri Bhandar | 521428 | 3266686 | 1891 |
| Bajhang | 3 | 38 | Chiuri Bhandar | 521642 | 3266395 | 1968 |
| Bajhang | 4 | 39 | Chiuri Bhandar | 521550 | 3266736 | 1984 |
| Bajhang | 5 | 40 | Chiuri Bhandar | 521453 | 3266740 | 1901 |
| Bajhang | 1 | 41 | Nauli Nabodaya | 513961 | 3263601 | 1580 |
| Bajhang | 2 | 42 | Nauli Nabodaya | 514861 | 3263101 | 1550 |
| Bajhang | 3 | 43 | Nauli Nabodaya | 515161 | 3263001 | 1565 |
| Bajhang | 4 | 44 | Nauli Nabodaya | 515561 | 3263001 | 1580 |
| Bara | 1 | 45 | Haraiya | 311069 | 2998189 | 102 |
| Bara | 2 | 46 | Haraiya | 311169 | 2998339 | 102 |
| Bara | 3 | 47 | Haraiya | 311169 | 2998539 | 100 |
| Bara | 1 | 48 | Kachdiya Proposed CF | 317074 | 3009775 | 146 |
| Bara | 2 | 49 | Kachdiya Proposed CF | 316974 | 3009875 | 146 |
| Bara | 3 | 50 | Kachdiya Proposed CF | 316874 | 3010075 | 147 |
| Bara | 4 | 51 | Kachdiya Proposed CF | 317074 | 3009975 | 146 |
| Bara | 1 | 52 | Kakadi VDC | 314550 | 2999770 | 108 |
| Bara | 2 | 53 | Kakadi VDC | 314550 | 2999820 | 106 |
| Bara | 3 | 54 | Kakadi VDC | 314550 | 3000020 | 105 |
| Bara | 4 | 55 | Kakadi VDC | 314350 | 3000120 | 105 |
| Bara | 1 | 56 | Setidevi Forest Management and Conservation Committee | 314525 | 3003931 | 150 |
| Bara | 2 | 57 | Setidevi Forest Management and Conservation Committee | 314285 | 3003991 | 149 |
| Bara | 3 | 58 | Setidevi Forest Management and Conservation Committee | 314105 | 3003931 | 149 |
| Bara | 4 | 59 | Setidevi Forest Management and Conservation Committee | 313745 | 3003811 | 149 |
| Bara | 1 | 60 | Shree Bramha Baba CF | 327795 | 3009464 | 208 |
| Bara | 2 | 61 | Shree Bramha Baba CF | 327995 | 3009565 | 209 |
| Bara | 3 | 62 | Shree Bramha Baba CF | 328195 | 3009864 | 209 |
| Bara | 4 | 63 | Shree Bramha Baba CF | 328195 | 3010164 | 211 |
| Bara | 5 | 64 | Shree Bramha Baba CF | 328495 | 3010164 | 211 |
| Bardia | 1 | 65 | Samjhana | 522074 | 3157550 | 177 |
| Bardia | 2 | 66 | Samjhana | 522174 | 3157806 | 176 |
| Bardia | 3 | 67 | Samjhana | 522274 | 3157906 | 176 |
| Bardia | 1 | 68 | Shree Kalika | 534160 | 3133164 | 145 |
| Bardia | 2 | 69 | Shree Kalika | 534155 | 3133588 | 146 |
| Bardia | 3 | 70 | Shree Kalika | 533953 | 3133573 | 149 |
| Bardia | 4 | 71 | Shree Kalika | 534350 | 3133977 | 128 |
| Bardia | 5 | 72 | Shree Kalika | 534757 | 3134172 | 145 |
| Bhojpur | 1 | 73 | Kaijale | 510920 | 3013171 | 1240 |
| Bhojpur | 2 | 74 | Kaijale | 510826 | 3019857 | 1054 |
| Bhojpur | 3 | 75 | Kaijale | 511504 | 3018030 | 1044 |
| Bhojpur | 1 | 76 | Salley | 510935 | 3016129 | 1221 |
| Bhojpur | 2 | 77 | Salley | 511106 | 3016284 | 1182 |
| Bhojpur | 3 | 78 | Salley | 511098 | 3016340 | 1192 |
| Chitwan | 1 | 79 | Ajingare | 262645 | 3063329 | 401 |
| Chitwan | 2 | 80 | Ajingare | 263045 | 3063529 | 367 |
| Chitwan | 3 | 81 | Ajingare | 263245 | 3063729 | 340 |
| Chitwan | 4 | 82 | Ajingare | 263445 | 3064129 | 421 |
| Chitwan | 5 | 83 | Ajingare | 262445 | 3063929 | 542 |
| Chitwan | 6 | 84 | Ajingare | 262245 | 3064129 | 607 |
| Chitwan | 7 | 85 | Ajingare | 263245 | 3064529 | 582 |
| Chitwan | 1 | 86 | Amrit Dharapani | 263376 | 3060148 | 323 |
| Chitwan | 2 | 87 | Amrit Dharapani | 263376 | 3031348 | 263 |
| Chitwan | 3 | 88 | Amrit Dharapani | 264176 | 3064184 | 445 |
| Chitwan | 4 | 89 | Amrit Dharapani | 264176 | 3063748 | 434 |
| Chitwan | 5 | 90 | Amrit Dharapani | 263776 | 3061748 | 302 |
| Chitwan | 6 | 91 | Amrit Dharapani | 263376 | 3062948 | 314 |
| Chitwan | 7 | 92 | Amrit Dharapani | 264576 | 3062948 | 294 |
| Dailekh | 1 | 93 | Kalika | 546333 | 3261280 | 2775 |
| Dailekh | 2 | 94 | Kalika | 546704 | 3199929 | 1029 |
| Dailekh | 3 | 95 | Kalika | 546716 | 3201103 | 966 |
| Dailekh | 4 | 96 | Kalika | 546700 | 3200457 | 1023 |
| Dailekh | 5 | 97 | Kalika | 546520 | 3200441 | 1066 |
| Dailekh | 6 | 98 | Kalika | 546215 | 3200886 | 1101 |
| Dang | 1 | 99 | Janashakti | 375282 | 3077012 | 296 |
| Dang | 2 | 100 | Janashakti | 375281 | 3076212 | 326 |
| Dang | 3 | 101 | Janashakti | 375110 | 3077736 | 282 |
| Dang | 4 | 102 | Janashakti | 374732 | 3076812 | 294 |
| Dang | 5 | 103 | Janashakti | 374582 | 3076412 | 302 |
| Dang | 6 | 104 | Janashakti | 374082 | 3075612 | 312 |
| Dang | 7 | 105 | Janashakti | 375322 | 3076017 | 310 |
| Dang | 1 | 106 | Kalikadevi | 649541 | 3104867 | 872 |
| Dang | 2 | 107 | Kalikadevi | 648941 | 3104867 | 902 |
| Dang | 3 | 108 | Kalikadevi | 648741 | 3104867 | 928 |
| Dang | 4 | 109 | Kalikadevi | 648356 | 3104778 | 996 |
| Dang | 5 | 110 | Kalikadevi | 648441 | 3104267 | 734 |
| Dang | 1 | 111 | Mahila Jagriti | 375110 | 3078086 | 297 |
| Dang | 2 | 112 | Mahila Jagriti | 375110 | 3077886 | 292 |
| Dang | 3 | 113 | Mahila Jagriti | 375110 | 3077736 | 282 |
| Dang | 4 | 114 | Mahila Jagriti | 375110 | 3077686 | 287 |
| Dang | 1 | 115 | Sawarikot | 356532 | 3101587 | 936 |
| Dang | 2 | 116 | Sawarikot | 356543 | 3101153 | 823 |
| Dang | 3 | 117 | Sawarikot | 356143 | 3101152 | 789 |
| Dang | 4 | 118 | Sawarikot | 356543 | 3102552 | 1151 |
| Dang | 5 | 119 | Sawarikot | 356543 | 3102552 | 1207 |
| Dang | 6 | 120 | Sawarikot | 356856 | 3102680 | 1243 |
| Dang | 1 | 121 | Sunsari | 639935 | 3114431 | 1252 |
| Dang | 2 | 122 | Sunsari | 640367 | 3114294 | 1296 |
| Dang | 3 | 123 | Sunsari | 640505 | 3114031 | 1269 |
| Dang | 4 | 124 | Sunsari | 640347 | 3114002 | 1192 |
| Darchula | 1 | 125 | Sewakendra | 442767 | 3281308 | 610 |
| Darchula | 2 | 126 | Sewakendra | 442867 | 3281308 | 619 |
| Darchula | 3 | 127 | Sewakendra | 442867 | 3281508 | 611 |
| Dhading | 1 | 128 | Betini | 312123 | 3073655 | 907 |
| Dhading | 2 | 129 | Betini | 312203 | 3073655 | 902 |
| Dhading | 3 | 130 | Betini | 312163 | 3073735 | 938 |
| Dhading | 1 | 131 | Dumla | 296531 | 3016697 | 1850 |
| Dhading | 2 | 132 | Dumla | 296556 | 3016747 | 1873 |
| Dhading | 3 | 133 | Dumla | 296656 | 3106897 | 1881 |
| Dhading | 4 | 134 | Dumla | 296656 | 3106847 | 1869 |
| Dhading | 5 | 135 | Dumla | 296606 | 3108647 | 1854 |
| Dhading | 1 | 136 | Takmare | 396094 | 3106654 | 1688 |
| Dhading | 3 | 137 | Takmare | 295697 | 3106354 | 1150 |
| Dhading | 4 | 138 | Takmare | 295794 | 3106454 | 1540 |
| Dhankuta | 1 | 139 | Chetmala CF | 529896 | 2973016 | 1044 |
| Dhankuta | 2 | 140 | Chetmala CF | 529286 | 2973016 | 1407 |
| Dhankuta | 3 | 141 | Chetmala CF | 528986 | 2973016 | 1181 |
| Dhankuta | 4 | 142 | Chetmala CF | 528986 | 2972416 | 1164 |
| Dhankuta | 5 | 143 | Chetmala CF | 528686 | 2972716 | 1230 |
| Dhankuta | 1 | 144 | Dharmasala CF | 528746 | 2986409 | 1768 |
| Dhankuta | 2 | 145 | Dharmasala CF | 528777 | 2986410 | 1788 |
| Dhankuta | 3 | 146 | Dharmasala CF | 528807 | 2986440 | 1755 |
| Dhankuta | 1 | 147 | Saj Bote NF | 540876 | 2977427 | 1048 |
| Dhankuta | 2 | 148 | Saj Bote NF | 540801 | 2977127 | 1039 |
| Dhankuta | 3 | 149 | Saj Bote NF | 540876 | 2977127 | 1111 |
| Dhankuta | 4 | 150 | Saj Bote NF | 540801 | 2976977 | 1064 |
| Dhankuta | 5 | 151 | Saj Bote NF | 540951 | 2976827 | 1132 |
| Dolakha | 1 | 152 | Dimal | 398582 | 3061020 | 1777 |
| Dolakha | 2 | 153 | Dimal | 398332 | 3061120 | 1751 |
| Dolakha | 3 | 154 | Dimal | 398282 | 3061170 | 1770 |
| Dolakha | 4 | 155 | Dimal | 397932 | 3061270 | 1769 |
| Dolakha | 1 | 156 | Gumbamahabir | 405706 | 3063925 | 1661 |
| Dolakha | 2 | 157 | Gumbamahabir | 406753 | 3063182 | 1722 |
| Dolakha | 3 | 158 | Gumbamahabir | 406239 | 3063178 | 1738 |
| Dolakha | 4 | 159 | Gumbamahabir | 405303 | 3063182 | 2201 |
| Dolakha | 5 | 160 | Gumbamahabir | 405269 | 3062984 | 2196 |
| Dolakha | 6 | 161 | Gumbamahabir | 406328 | 3063297 | 1659 |
| Dolakha | 1 | 162 | Khorthali | 405098 | 3063472 | 2086 |
| Dolakha | 2 | 163 | Khorthali | 405298 | 3033072 | 2232 |
| Dolakha | 3 | 164 | Khorthali | 405498 | 3061872 | 2355 |
| Dolakha | 4 | 165 | Khorthali | 405498 | 3061472 | 2321 |
| Dolakha | 5 | 166 | Khorthali | 404598 | 3062872 | 2200 |
| Dolakha | 6 | 167 | Khorthali | 404598 | 3063072 | 2110 |
| Dolakha | 1 | 168 | Patiko Ban | 397191 | 3066220 | 2465 |
| Dolakha | 2 | 169 | Patiko Ban | 397296 | 3066114 | 2453 |
| Dolakha | 3 | 170 | Patiko Ban | 397322 | 3065967 | 2421 |
| Dolakha | 4 | 171 | Patiko Ban | 397264 | 3065888 | 2366 |
| Dolakha | 5 | 172 | Patiko Ban | 397233 | 3065929 | 2348 |
| Doti | 1 | 173 | Kalika | 487781 | 3246756 | 1489 |
| Doti | 2 | 174 | Kalika | 488531 | 3246956 | 1510 |
| Doti | 3 | 175 | Kalika | 488131 | 3246756 | 1502 |
| Doti | 1 | 176 | Punyapati | 494665 | 3240125 | 1155 |
| Doti | 2 | 177 | Punyapati | 494365 | 3240225 | 1478 |
| Doti | 3 | 178 | Punyapati | 494765 | 3240525 | 1423 |
| Doti | 4 | 179 | Punyapati | 493765 | 3240025 | 1480 |
| Doti | 5 | 180 | Punyapati | 493565 | 3239325 | 1470 |
| Doti | 6 | 181 | Punyapati | 493685 | 3238845 | 1450 |
| Gorkha | 1 | 182 | Jalgire | 279157 | 3109694 | 1060 |
| Gorkha | 2 | 183 | Jalgire | 279207 | 3109494 | 1089 |
| Gorkha | 3 | 184 | Jalgire | 278857 | 3109294 | 1088 |
| Gorkha | 4 | 185 | Jalgire | 278757 | 3109294 | 1069 |
| Gorkha | 5 | 186 | Jalgire | 278957 | 3109294 | 1100 |
| Gulmi | 1 | 187 | Kwanke deurali | 703888 | 3114486 | 1779 |
| Gulmi | 2 | 188 | Kwanke deurali | 703788 | 3113686 | 1787 |
| Gulmi | 3 | 189 | Kwanke deurali | 703488 | 3112986 | 1894 |
| Gulmi | 4 | 190 | Kwanke deurali | 703788 | 3113086 | 1832 |
| Gulmi | 1 | 191 | Nawajyoti | 723721 | 3109831 | 1221 |
| Gulmi | 2 | 192 | Nawajyoti | 723721 | 3110331 | 1327 |
| Gulmi | 3 | 193 | Nawajyoti | 722971 | 3110081 | 1105 |
| Gulmi | 4 | 194 | Nawajyoti | 723221 | 3110331 | 1131 |
| Gulmi | 5 | 195 | Nawajyoti | 723471 | 3110831 | 1359 |
| Gulmi | 6 | 196 | Nawajyoti | 722471 | 3111131 | 1045 |
| Gulmi | 7 | 197 | Nawajyoti | 722721 | 3110831 | 1141 |
| Gulmi | 1 | 198 | Thuloban | 738619 | 3092930 | 544 |
| Gulmi | 1 | 199 | Thuloban | 738519 | 3092933 | 603 |
| Gulmi | 1 | 200 | Thuloban | 738569 | 3092883 | 548 |
| Ilam | 1 | 201 | Bhawana | 583959 | 2956031 | 250 |
| Ilam | 2 | 202 | Bhawana | 583319 | 2956351 | 255 |
| Ilam | 3 | 203 | Bhawana | 582679 | 2656351 | 248 |
| Ilam | 4 | 204 | Bhawana | 582359 | 2955711 | 240 |
| Ilam | 5 | 205 | Bhawana | 581719 | 2955391 | 252 |
| Ilam | 6 | 206 | Bhawana | 582679 | 2956031 | 250 |
| Ilam | 7 | 207 | Bhawana | 583319 | 2956031 | 251 |
| Ilam | 1 | 208 | Saraswati | 585541 | 2956651 | 224 |
| Ilam | 2 | 209 | Saraswati | 585841 | 2954815 | 201 |
| Ilam | 3 | 210 | Saraswati | 585841 | 2955751 | 237 |
| Ilam | 4 | 211 | Saraswati | 585841 | 2955151 | 208 |
| Ilam | 5 | 212 | Saraswati | 586141 | 2954851 | 227 |
| Ilam | 6 | 213 | Saraswati | 586141 | 2955751 | 204 |
| Ilam | 7 | 214 | Saraswati | 586141 | 2955451 | 225 |
| Jhapa | 1 | 215 | Avimukteswor | 597684 | 2926092 | 49 |
| Jhapa | 2 | 216 | Avimukteswor | 597384 | 2926392 | 58 |
| Jhapa | 3 | 217 | Avimukteswor | 597084 | 2926992 | 75 |
| Jhapa | 4 | 218 | Avimukteswor | 597684 | 2927292 | 72 |
| Jhapa | 5 | 219 | Avimukteswor | 597384 | 2927592 | 56 |
| Jhapa | 6 | 220 | Avimukteswor | 597684 | 2928192 | 58 |
| Jhapa | 1 | 221 | Durga Bhitta | 598285 | 2927425 | 66 |
| Jhapa | 2 | 222 | Durga Bhitta | 598480 | 2927685 | 68 |
| Jhapa | 3 | 223 | Durga Bhitta | 598780 | 2927685 | 88 |
| Jhapa | 4 | 224 | Durga Bhitta | 598740 | 2927425 | 66 |
| Jhapa | 5 | 225 | Durga Bhitta | 598480 | 2927165 | 90 |
| Jhapa | 6 | 226 | Durga Bhitta | 598480 | 2928465 | 98 |
| Jhapa | 1 | 227 | Pragati | 604299 | 2948993 | 125 |
| Jhapa | 2 | 228 | Pragati | 603999 | 2948393 | 120 |
| Jhapa | 3 | 229 | Pragati | 603499 | 2948333 | 122 |
| Jhapa | 4 | 230 | Pragati | 602899 | 2948393 | 125 |
| Jhapa | 5 | 231 | Pragati | 603433 | 2948793 | 118 |
| Jhapa | 1 | 232 | Tribeni | 596828 | 2925979 | 98 |
| Jhapa | 2 | 233 | Tribeni | 596698 | 2926759 | 95 |
| Jhapa | 3 | 234 | Tribeni | 536698 | 2927019 | 72 |
| Jhapa | 4 | 235 | Tribeni | 536698 | 2927539 | 78 |
| Jhapa | 5 | 236 | Tribeni | 596698 | 2926439 | 88 |
| Kailali | 1 | 237 | Gwaladeu NF | 466596 | 3184663 | 188 |
| Kailali | 2 | 238 | Gwaladeu NF | 466496 | 3184463 | 188 |
| Kailali | 3 | 239 | Gwaladeu NF | 466296 | 3184263 | 188 |
| Kailali | 4 | 240 | Gwaladeu NF | 466396 | 3184163 | 188 |
| Kailali | 1 | 241 | Gwasi Samaichi | 465465 | 3184356 | 214 |
| Kailali | 2 | 242 | Gwasi Samaichi | 465165 | 3184256 | 216 |
| Kailali | 3 | 243 | Gwasi Samaichi | 464865 | 3184456 | 215 |
| Kailali | 4 | 244 | Gwasi Samaichi | 464285 | 3184216 | 208 |
| Kailali | 5 | 245 | Gwasi Samaichi | 465095 | 3183596 | 195 |
| Kailali | 1 | 246 | Kalika NF | 470136 | 3172178 | 168 |
| Kailali | 2 | 247 | Kalika NF | 470236 | 3172378 | 165 |
| Kailali | 3 | 248 | Kalika NF | 469936 | 3172478 | 166 |
| Kailali | 4 | 249 | Kalika NF | 470036 | 3172678 | 165 |
| Kailali | 1 | 250 | Laliguras NF | 477355 | 3174071 | 173 |
| Kailali | 2 | 251 | Laliguras NF | 477855 | 3175571 | 170 |
| Kailali | 3 | 252 | Laliguras NF | 478855 | 3175771 | 171 |
| Kailali | 4 | 253 | Laliguras NF | 478455 | 3176171 | 173 |
| Kailali | 5 | 254 | Laliguras NF | 479255 | 3176571 | 168 |
| Kailali | 6 | 255 | Laliguras NF | 478055 | 3176771 | 169 |
| Kailali | 7 | 256 | Laliguras NF | 478655 | 3176971 | 171 |
| Kailali | 1 | 257 | Maya Samaichi | 467289 | 3186039 | 170 |
| Kailali | 2 | 258 | Maya Samaichi | 467364 | 3186114 | 175 |
| Kailali | 3 | 259 | Maya Samaichi | 467314 | 3186088 | 178 |
| Kailali | 1 | 260 | Samaichi NF | 467277 | 3186391 | 170 |
| Kailali | 2 | 261 | Samaichi NF | 467477 | 3186391 | 170 |
| Kailali | 3 | 262 | Samaichi NF | 467277 | 3186491 | 169 |
| Kailali | 4 | 263 | Samaichi NF | 467377 | 3186691 | 180 |
| Kailali | 1 | 264 | Sannikot NF | 466858 | 3186264 | 229 |
| Kailali | 2 | 265 | Sannikot NF | 466658 | 3186464 | 236 |
| Kailali | 3 | 266 | Sannikot NF | 466858 | 3186464 | 233 |
| Kailali | 4 | 267 | Sannikot NF | 466554 | 3186364 | 234 |
| Kailali | 1 | 268 | Shiva Samaichi NCF | 465888 | 3186316 | 227 |
| Kailali | 2 | 269 | Shiva Samaichi NCF | 465988 | 3186316 | 227 |
| Kailali | 3 | 270 | Shiva Samaichi NCF | 465963 | 3186291 | 226 |
| Kanchanpur | 1 | 271 | Amar | 424273 | 3208853 | 268 |
| Kanchanpur | 2 | 272 | Amar | 424718 | 3209013 | 272 |
| Kanchanpur | 3 | 273 | Amar | 424835 | 3209213 | 272 |
| Kanchanpur | 4 | 274 | Amar | 424239 | 3209399 | 241 |
| Kanchanpur | 5 | 275 | Amar | 424039 | 3209199 | 255 |
| Kanchanpur | 6 | 276 | Amar | 423639 | 3208799 | 265 |
| Kanchanpur | 7 | 277 | Amar | 424239 | 3209799 | 230 |
| Kanchanpur | 1 | 278 | Bandevi | 448219 | 3190059 | 171 |
| Kanchanpur | 2 | 279 | Bandevi | 448119 | 3189859 | 171 |
| Kanchanpur | 3 | 280 | Bandevi | 448319 | 3189659 | 171 |
| Kanchanpur | 4 | 281 | Bandevi | 447819 | 3189759 | 169 |
| Kanchanpur | 5 | 282 | Bandevi | 447719 | 3189359 | 172 |
| Kanchanpur | 6 | 283 | Bandevi | 448219 | 3189159 | 171 |
| Kanchanpur | 1 | 284 | Sita | 454204 | 3176183 | 192 |
| Kanchanpur | 2 | 285 | Sita | 453904 | 3176383 | 195 |
| Kanchanpur | 3 | 286 | Sita | 453704 | 3176883 | 199 |
| Kanchanpur | 4 | 287 | Sita | 454104 | 3177183 | 194 |
| Kanchanpur | 5 | 288 | Sita | 454204 | 3176883 | 195 |
| Kapilvastu | 1 | 289 | Badaganga | 715676 | 3067209 | 151 |
| Kapilvastu | 2 | 290 | Badaganga | 715551 | 3067011 | 150 |
| Kapilvastu | 3 | 291 | Badaganga | 715451 | 3066836 | 152 |
| Kapilvastu | 4 | 292 | Badaganga | 715376 | 3066686 | 150 |
| Kapilvastu | 1 | 293 | Bankasbasha NF | 682956 | 3069015 | 182 |
| Kapilvastu | 2 | 294 | Bankasbasha NF | 682956 | 3068640 | 158 |
| Kapilvastu | 3 | 295 | Bankasbasha NF | 683406 | 3068790 | 155 |
| Kapilvastu | 4 | 296 | Bankasbasha NF | 682281 | 3068790 | 219 |
| Kapilvastu | 5 | 297 | Bankasbasha NF | 682491 | 3069105 | 180 |
| Kapilvastu | 6 | 298 | Bankasbasha NF | 682956 | 3068115 | 166 |
| Kapilvastu | 7 | 299 | Bankasbasha NF | 682041 | 3068355 | 167 |
| Kapilvastu | 1 | 300 | Googauli NF | 674331 | 3060264 | 141 |
| Kapilvastu | 2 | 301 | Googauli NF | 674230 | 3060086 | 131 |
| Kapilvastu | 3 | 302 | Googauli NF | 674032 | 3060331 | 112 |
| Kapilvastu | 4 | 303 | Googauli NF | 673932 | 3059931 | 130 |
| Kapilvastu | 5 | 304 | Googauli NF | 673832 | 3060131 | 118 |
| Kapilvastu | 1 | 305 | Panbari NF | 703398 | 3069338 | 176 |
| Kapilvastu | 2 | 306 | Panbari NF | 703248 | 3069188 | 178 |
| Kapilvastu | 3 | 307 | Panbari NF | 702948 | 3069338 | 174 |
| Kapilvastu | 4 | 308 | Panbari NF | 702348 | 3069338 | 184 |
| Kapilvastu | 5 | 309 | Panbari NF | 702198 | 3069488 | 182 |
| Kapilvastu | 6 | 310 | Panbari NF | 701748 | 3069488 | 181 |
| Kapilvastu | 7 | 311 | Panbari NF | 701748 | 3069188 | 179 |
| Kapilvastu | 1 | 312 | Sringighat NCF | 716706 | 3070903 | 108 |
| Kapilvastu | 2 | 313 | Sringighat NCF | 716033 | 3070582 | 104 |
| Kapilvastu | 3 | 314 | Sringighat NCF | 716326 | 3070302 | 110 |
| Kapilvastu | 4 | 315 | Sringighat NCF | 716726 | 3070313 | 110 |
| Kapilvastu | 5 | 316 | Sringighat NCF | 715126 | 3070312 | 111 |
| Kapilvastu | 6 | 317 | Sringighat NCF | 715746 | 3070902 | 113 |
| Kapilvastu | 7 | 318 | Sringighat NCF | 716032 | 3069687 | 109 |
| Kaski | 1 | 319 | Baunaekoti lek jahare banpale kalchepari | 787496 | 3119974 | 1124 |
| Kaski | 2 | 320 | Baunaekoti lek jahare banpale kalchepari | 787586 | 3119614 | 1241 |
| Kaski | 3 | 321 | Baunaekoti lek jahare banpale kalchepari | 788036 | 3119254 | 1094 |
| Kaski | 4 | 322 | Baunaekoti lek jahare banpale kalchepari | 788126 | 3119254 | 1076 |
| Kaski | 1 | 323 | Dandapari | 789115 | 3128805 | 1254 |
| Kaski | 2 | 324 | Dandapari | 788915 | 3129005 | 1354 |
| Kaski | 3 | 325 | Dandapari | 788712 | 3129205 | 1485 |
| Kaski | 4 | 326 | Dandapari | 788515 | 3129505 | 1416 |
| Kaski | 5 | 327 | Dandapari | 788415 | 3129705 | 1472 |
| Kaski | 1 | 328 | Kalane | 787237 | 3120352 | 885 |
| Kaski | 3 | 329 | Kalane | 787287 | 3120302 | 885 |
| Kaski | 2 | 330 | Kalane | 787187 | 3120202 | 885 |
| Kaski | 1 | 331 | Khalte khola | 791586 | 3117303 | 851 |
| Kaski | 2 | 332 | Khalte khola | 791786 | 3117103 | 810 |
| Kaski | 3 | 333 | Khalte khola | 792086 | 3117303 | 820 |
| Kaski | 4 | 334 | Khalte khola | 791986 | 3117503 | 950 |
| Kaski | 1 | 335 | Saderani | 787478 | 3129441 | 1624 |
| Kaski | 2 | 336 | Saderani | 787378 | 3129541 | 1636 |
| Kaski | 3 | 337 | Saderani | 787178 | 3129641 | 1604 |
| Kaski | 4 | 338 | Saderani | 787178 | 3129841 | 1650 |
| Kavrepalanchok | 1 | 339 | Dhobidhara | 352245 | 3058637 | 1645 |
| Kavrepalanchok | 2 | 340 | Dhobidhara | 352275 | 3058642 | 1685 |
| Kavrepalanchok | 3 | 341 | Dhobidhara | 352275 | 3058632 | 1609 |
| Kavrepalanchok | 3 | 342 | Tattentar | 364431 | 3049654 | 1265 |
| Kavrepalanchok | 2 | 343 | Tattentar | 364271 | 3049814 | 1234 |
| Kavrepalanchok | 1 | 344 | Tattentar | 364111 | 3049854 | 1261 |
| Mahottari | 1 | 345 | Sagarnath F.mgmt Project | 381512 | 2983458 | 134 |
| Mahottari | 2 | 346 | Sagarnath F.mgmt Project | 381312 | 2983558 | 140 |
| Mahottari | 3 | 347 | Sagarnath F.mgmt Project | 381312 | 2983758 | 140 |
| Mahottari | 4 | 348 | Sagarnath F.mgmt Project | 381412 | 2983958 | 142 |
| Mahottari | 1 | 349 | Srijana | 380584 | 2983677 | 147 |
| Mahottari | 2 | 350 | Srijana | 380677 | 2983680 | 147 |
| Mahottari | 3 | 351 | Srijana | 380783 | 2983676 | 137 |
| Makwanpur | 1 | 352 | Mendoling | 884601 | 3049741 | 552 |
| Makwanpur | 2 | 353 | Mendoling | 885101 | 3048741 | 764 |
| Makwanpur | 3 | 354 | Mendoling | 885601 | 3048491 | 845 |
| Makwanpur | 4 | 355 | Mendoling | 886351 | 3047991 | 881 |
| Makwanpur | 5 | 356 | Mendoling | 887101 | 3048741 | 522 |
| Makwanpur | 6 | 357 | Mendoling | 886351 | 3049241 | 582 |
| Makwanpur | 7 | 358 | Mendoling | 885851 | 3039741 | 492 |
| Morang | 1 | 359 | Srijana Bhaunne | 546870 | 2948131 | 151 |
| Morang | 2 | 360 | Srijana Bhaunne | 547110 | 2948491 | 156 |
| Morang | 3 | 361 | Srijana Bhaunne | 547110 | 2948671 | 158 |
| Morang | 4 | 362 | Srijana Bhaunne | 546790 | 2948581 | 156 |
| Morang | 1 | 363 | Srijana Gothgaun | 534515 | 2951628 | 152 |
| Morang | 2 | 364 | Srijana Gothgaun | 535115 | 2952028 | 160 |
| Morang | 3 | 365 | Srijana Gothgaun | 535115 | 2952428 | 160 |
| Morang | 4 | 366 | Srijana Gothgaun | 535415 | 2952828 | 170 |
| Morang | 5 | 367 | Srijana Gothgaun | 536015 | 2953228 | 172 |
| Morang | 6 | 368 | Srijana Gothgaun | 536015 | 2953628 | 178 |
| Morang | 7 | 369 | Srijana Gothgaun | 536315 | 2954028 | 181 |
| Myagdi | 1 | 370 | Pauleya ashram githini | 750104 | 3139337 | 1554 |
| Myagdi | 2 | 371 | Pauleya ashram githini | 750220 | 3139196 | 1471 |
| Myagdi | 3 | 372 | Pauleya ashram githini | 750007 | 3139098 | 1458 |
| Myagdi | 4 | 373 | Pauleya ashram githini | 749742 | 3138892 | 1435 |
| Myagdi | 1 | 374 | RanibanA | 436264 | 3154527 | 1830 |
| Myagdi | 2 | 375 | RanibanA | 436064 | 3154627 | 1977 |
| Myagdi | 3 | 376 | RanibanA | 436064 | 3154827 | 2046 |
| Myagdi | 4 | 377 | RanibanA | 435464 | 3155127 | 2000 |
| Myagdi | 5 | 378 | RanibanA | 435464 | 3155427 | 2000 |
| Nawalparasi | 1 | 379 | Ankur | 213164 | 3060422 | 179 |
| Nawalparasi | 2 | 380 | Ankur | 213164 | 3061222 | 190 |
| Nawalparasi | 3 | 381 | Ankur | 213164 | 3061922 | 198 |
| Nawalparasi | 4 | 382 | Ankur | 212764 | 3061222 | 199 |
| Nawalparasi | 5 | 383 | Ankur | 212764 | 3061922 | 205 |
| Nawalparasi | 1 | 384 | Mayur Pokhari | 210799 | 3061156 | 219 |
| Nawalparasi | 2 | 385 | Mayur Pokhari | 210199 | 3062356 | 242 |
| Nawalparasi | 3 | 386 | Mayur Pokhari | 210799 | 3061556 | 238 |
| Nawalparasi | 4 | 387 | Mayur Pokhari | 210799 | 3061956 | 235 |
| Nawalparasi | 5 | 388 | Mayur Pokhari | 209999 | 3063156 | 243 |
| Nawalparasi | 1 | 389 | Milijuli | 213178 | 3060039 | 170 |
| Nawalparasi | 2 | 390 | Milijuli | 212707 | 3059839 | 165 |
| Nawalparasi | 3 | 391 | Milijuli | 212878 | 3059839 | 165 |
| Nawalparasi | 1 | 392 | Srijhansil Kha | 212393 | 3059215 | 175 |
| Nawalparasi | 2 | 393 | Srijhansil Kha | 212593 | 3059315 | 178 |
| Nawalparasi | 3 | 394 | Srijhansil Kha | 212493 | 3060115 | 177 |
| Nawalparasi | 4 | 395 | Srijhansil Kha | 212493 | 3060315 | 180 |
| Nawalparasi | 1 | 396 | Trikon | 211665 | 3063056 | 218 |
| Nawalparasi | 2 | 397 | Trikon | 211665 | 3062256 | 219 |
| Nawalparasi | 3 | 398 | Trikon | 211665 | 3062656 | 197 |
| Nawalparasi | 4 | 399 | Trikon | 211275 | 3062256 | 212 |
| Nawalparasi | 5 | 400 | Trikon | 211275 | 3061456 | 215 |
| Palpa | 1 | 401 | Salleri Rajbrikshya CF | 746256 | 3079793 | 1228 |
| Palpa | 2 | 402 | Salleri Rajbrikshya CF | 746250 | 3079613 | 1236 |
| Palpa | 3 | 403 | Salleri Rajbrikshya CF | 746190 | 3079010 | 1102 |
| Palpa | 4 | 404 | Salleri Rajbrikshya CF | 745830 | 3079193 | 1110 |
| Palpa | 1 | 405 | Sukadamar NF | 725597 | 3075493 | 912 |
| Palpa | 2 | 406 | Sukadamar NF | 726220 | 3075691 | 845 |
| Palpa | 3 | 407 | Sukadamar NF | 726320 | 3075991 | 918 |
| Palpa | 4 | 408 | Sukadamar NF | 726220 | 3075591 | 907 |
| Pamechhap | 1 | 409 | Bhumethan Salyan | 385796 | 3040567 | 1544 |
| Pamechhap | 2 | 410 | Bhumethan Salyan | 386021 | 3040417 | 1522 |
| Pamechhap | 3 | 411 | Bhumethan Salyan | 386321 | 3040492 | 1602 |
| Pamechhap | 4 | 412 | Bhumethan Salyan | 386246 | 3040567 | 1562 |
| Pamechhap | 5 | 413 | Bhumethan Salyan | 385496 | 3040792 | 1502 |
| Pamechhap | 1 | 414 | Simpuri Tilkhola | 401610 | 3029297 | 602 |
| Pamechhap | 2 | 415 | Simpuri Tilkhola | 401660 | 3029147 | 590 |
| Pamechhap | 3 | 416 | Simpuri Tilkhola | 401810 | 3028997 | 504 |
| Pamechhap | 4 | 417 | Simpuri Tilkhola | 401910 | 3029497 | 530 |
| Pamechhap | 5 | 418 | Simpuri Tilkhola | 401960 | 3029397 | 596 |
| Parbat | 1 | 419 | Damaha dhunga | 764159 | 3113731 | 1799 |
| Parbat | 2 | 420 | Damaha dhunga | 764209 | 3113781 | 1842 |
| Parbat | 3 | 421 | Damaha dhunga | 764309 | 3113731 | 1893 |
| Parbat | 1 | 422 | Kaligandaki | 760936 | 3127064 | 879 |
| Parbat | 2 | 423 | Kaligandaki | 761519 | 3126986 | 1089 |
| Parbat | 3 | 424 | Kaligandaki | 761940 | 3126553 | 1081 |
| Parbat | 4 | 425 | Kaligandaki | 761836 | 3126357 | 994 |
| Parbat | 5 | 426 | Kaligandaki | 761626 | 3127164 | 1011 |
| Parbat | 1 | 427 | Thulo Salleri | 753333 | 3139289 | 1786 |
| Parbat | 2 | 428 | Thulo Salleri | 753166 | 3139348 | 1697 |
| Parbat | 3 | 429 | Thulo Salleri | 753026 | 3139239 | 1598 |
| Parbat | 4 | 430 | Thulo Salleri | 752913 | 3139479 | 1576 |
| Parsa | 1 | 431 | Shikar | 283648 | 2995584 | 89 |
| Parsa | 2 | 432 | Shikar | 283640 | 2996064 | 91 |
| Parsa | 3 | 433 | Shikar | 283707 | 2996499 | 91 |
| Pyuthan | 1 | 434 | Mallarani Dhaichaur | 686919 | 3107279 | 1386 |
| Pyuthan | 2 | 435 | Mallarani Dhaichaur | 686819 | 3107479 | 1398 |
| Pyuthan | 3 | 436 | Mallarani Dhaichaur | 686919 | 3107279 | 1367 |
| Pyuthan | 4 | 437 | Mallarani Dhaichaur | 686019 | 3107179 | 1348 |
| Pyuthan | 5 | 438 | Mallarani Dhaichaur | 686019 | 3107179 | 1322 |
| Pyuthan | 1 | 439 | Salleri Pakha | 659069 | 3114472 | 1721 |
| Pyuthan | 2 | 440 | Salleri Pakha | 658571 | 3116960 | 1558 |
| Pyuthan | 3 | 441 | Salleri Pakha | 658383 | 3117310 | 1503 |
| Pyuthan | 4 | 442 | Salleri Pakha | 658683 | 3117560 | 1529 |
| Rolpa | 1 | 443 | Baraha Chhetra | 663809 | 3123146 | 998 |
| Rolpa | 2 | 444 | Baraha Chhetra | 663609 | 3122946 | 950 |
| Rolpa | 3 | 445 | Baraha Chhetra | 663459 | 3107349 | 1172 |
| Rolpa | 4 | 446 | Baraha Chhetra | 663042 | 3123339 | 1196 |
| Rolpa | 5 | 447 | Baraha Chhetra | 663259 | 3123046 | 983 |
| Rolpa | 6 | 448 | Baraha Chhetra | 662349 | 3122048 | 988 |
| Rupandehi | 1 | 449 | Baunakoti | 741469 | 3051692 | 129 |
| Rupandehi | 2 | 450 | Baunakoti | 741468 | 3051293 | 96 |
| Rupandehi | 3 | 451 | Baunakoti | 741462 | 3050893 | 96 |
| Rupandehi | 4 | 452 | Baunakoti | 741671 | 3050890 | 93 |
| Rupandehi | 5 | 453 | Baunakoti | 742081 | 3051086 | 90 |
| Rupandehi | 6 | 454 | Baunakoti | 741467 | 3050692 | 90 |
| Rupandehi | 1 | 455 | Rohini NCF | 748776 | 3061758 | 140 |
| Rupandehi | 2 | 456 | Rohini NCF | 748476 | 3061758 | 132 |
| Rupandehi | 3 | 457 | Rohini NCF | 748576 | 3061958 | 125 |
| Rupandehi | 4 | 458 | Rohini NCF | 747976 | 3061958 | 128 |
| Rupandehi | 5 | 459 | Rohini NCF | 747776 | 3062158 | 130 |
| Rupandehi | 1 | 460 | Siktahan CF | 754476 | 3050574 | 115 |
| Rupandehi | 2 | 461 | Siktahan CF | 754416 | 3050518 | 112 |
| Rupandehi | 3 | 462 | Siktahan CF | 754416 | 3050698 | 116 |
| Rupandehi | 1 | 463 | Sukhaula Hariyali | 749350 | 3061572 | 129 |
| Rupandehi | 2 | 464 | Sukhaula Hariyali | 749550 | 3060872 | 124 |
| Rupandehi | 3 | 465 | Sukhaula Hariyali | 749650 | 3060672 | 123 |
| Salyan | 1 | 466 | Jyamire | 607057 | 3128038 | 1063 |
| Salyan | 2 | 467 | Jyamire | 607232 | 3128171 | 869 |
| Salyan | 3 | 468 | Jyamire | 607457 | 3128278 | 776 |
| Salyan | 4 | 469 | Jyamire | 607457 | 3128378 | 742 |
| Salyan | 1 | 470 | Laligurans | 626587 | 3143527 | 1542 |
| Salyan | 2 | 471 | Laligurans | 626632 | 3143241 | 1656 |
| Salyan | 3 | 472 | Laligurans | 626732 | 3143103 | 1598 |
| Salyan | 4 | 473 | Laligurans | 626616 | 3142932 | 1544 |
| Sarlahi | 1 | 474 | Loktantarik Namuna Proposed CF | 351438 | 2991449 | 104 |
| Sarlahi | 2 | 475 | Loktantarik Namuna Proposed CF | 351376 | 2991269 | 103 |
| Sarlahi | 3 | 476 | Loktantarik Namuna Proposed CF | 351258 | 2991089 | 106 |
| Sarlahi | 4 | 477 | Loktantarik Namuna Proposed CF | 351138 | 2990909 | 107 |
| Sindhuli | 1 | 478 | Bhiman Panesi | 396349 | 2999233 | 442 |
| Sindhuli | 2 | 479 | Bhiman Panesi | 395449 | 2999683 | 550 |
| Sindhuli | 3 | 480 | Bhiman Panesi | 395749 | 2999083 | 472 |
| Sindhuli | 4 | 481 | Bhiman Panesi | 395749 | 2998483 | 448 |
| Sindhuli | 5 | 482 | Bhiman Panesi | 396049 | 2998183 | 436 |
| Sindhuli | 6 | 483 | Bhiman Panesi | 395749 | 2997583 | 437 |
| Sindhuli | 7 | 484 | Bhiman Panesi | 396349 | 2997583 | 418 |
| Sindhuli | 1 | 485 | Indrawati | 695809 | 3004396 | 883 |
| Sindhuli | 2 | 486 | Indrawati | 695359 | 3004846 | 907 |
| Sindhuli | 3 | 487 | Indrawati | 695209 | 3005296 | 889 |
| Sindhuli | 4 | 488 | Indrawati | 695309 | 3005746 | 909 |
| Sindhuli | 5 | 489 | Indrawati | 695509 | 3005596 | 856 |
| Sindhuli | 6 | 490 | Indrawati | 695509 | 3005746 | 805 |
| Sindhuli | 7 | 491 | Indrawati | 694757 | 3004846 | 743 |
| Sindhuli | 1 | 492 | Kamala | 416397 | 2987334 | 270 |
| Sindhuli | 2 | 493 | Kamala | 416291 | 2986208 | 357 |
| Sindhuli | 3 | 494 | Kamala | 416778 | 2987025 | 271 |
| Sindhuli | 4 | 495 | Kamala | 417072 | 2987227 | 257 |
| Sindhuli | 5 | 496 | Kamala | 417068 | 2987426 | 247 |
| Sindhuli | 1 | 497 | Kusheswor Dumja | 383264 | 3033409 | 1167 |
| Sindhuli | 2 | 498 | Kusheswor Dumja | 383414 | 3033259 | 1165 |
| Sindhuli | 3 | 499 | Kusheswor Dumja | 383264 | 3032959 | 1224 |
| Sindhuli | 4 | 500 | Kusheswor Dumja | 382964 | 3032659 | 1232 |
| Sindhuli | 5 | 501 | Kusheswor Dumja | 382514 | 3032809 | 1066 |
| Sindhuli | 1 | 502 | Saleni Tarebhi | 379930 | 3032843 | 1274 |
| Sindhuli | 2 | 503 | Saleni Tarebhi | 380530 | 3032843 | 991 |
| Sindhuli | 3 | 504 | Saleni Tarebhi | 381330 | 3033043 | 870 |
| Sindhuli | 4 | 505 | Saleni Tarebhi | 381930 | 3033443 | 1027 |
| Sindhuli | 5 | 506 | Saleni Tarebhi | 381730 | 3034643 | 657 |
| Sindhuli | 6 | 507 | Saleni Tarebhi | 380730 | 3034243 | 1172 |
| Sindhuli | 1 | 508 | Shivashakti |  |  | 500 |
| Sindhuli | 2 | 509 | Shivashakti | 416300 | 2991567 | 412 |
| Sindhuli | 3 | 510 | Shivashakti | 416367 | 2992242 | 514 |
| Sindhuli | 4 | 511 | Shivashakti | 415714 | 2991255 | 437 |
| Sindhuli | 5 | 512 | Shivashakti | 416145 | 2990624 | 253 |
| Sindhuli | 6 | 513 | Shivashakti |  |  | 450 |
| Sindhupalchok | 1 | 514 | Channge khola | 354737 | 3086800 | 1356 |
| Sindhupalchok | 2 | 515 | Channge khola | 354837 | 3086900 | 1362 |
| Sindhupalchok | 3 | 516 | Channge khola | 354837 | 3087000 | 1392 |
| Sindhupalchok | 4 | 517 | Channge khola | 354687 | 3087100 | 1406 |
| Sindhupalchok | 5 | 518 | Channge khola | 354587 | 3087000 | 1362 |
| Surkhet | 1 | 519 | Bheri | 586514 | 3146174 | 673 |
| Surkhet | 2 | 520 | Bheri | 586489 | 3146049 | 690 |
| Surkhet | 3 | 521 | Bheri | 586264 | 3146516 | 710 |
| Surkhet | 4 | 522 | Bheri | 586934 | 3146234 | 680 |
| Surkhet | 1 | 523 | Bheri CF | 565395 | 3156969 | 659 |
| Surkhet | 2 | 524 | Bheri CF | 565643 | 3156644 | 620 |
| Surkhet | 3 | 525 | Bheri CF | 565662 | 3156353 | 597 |
| Surkhet | 4 | 526 | Bheri CF | 565819 | 3156157 | 568 |
| Surkhet | 5 | 527 | Bheri CF | 565643 | 3155625 | 544 |
| Surkhet | 6 | 528 | Bheri CF | 565719 | 3155236 | 496 |
| Surkhet | 7 | 529 | Bheri CF | 566131 | 3156094 | 508 |
| Surkhet | 1 | 530 | Deuti Proposed CF | 584759 | 3143029 | 980 |
| Surkhet | 2 | 531 | Deuti Proposed CF | 584881 | 3148774 | 1202 |
| Surkhet | 3 | 532 | Deuti Proposed CF | 585607 | 3142527 | 615 |
| Surkhet | 4 | 533 | Deuti Proposed CF | 586027 | 3142654 | 634 |
| Surkhet | 5 | 534 | Deuti Proposed CF | 586270 | 3143189 | 708 |
| Surkhet | 1 | 535 | Hariyali | 572074 | 3137459 | 682 |
| Surkhet | 2 | 536 | Hariyali | 591356 | 3138224 | 680 |
| Surkhet | 3 | 537 | Hariyali | 590722 | 3138560 | 695 |
| Surkhet | 4 | 538 | Hariyali | 591760 | 3138848 | 703 |
| Surkhet | 5 | 539 | Hariyali | 592280 | 3137938 | 645 |
| Surkhet | 1 | 540 | Pokharidanda CF | 566448 | 3161935 | 699 |
| Surkhet | 2 | 541 | Pokharidanda CF | 566396 | 3162177 | 678 |
| Surkhet | 3 | 542 | Pokharidanda CF | 566600 | 3162632 | 696 |
| Surkhet | 4 | 543 | Pokharidanda CF | 566739 | 3162226 | 758 |
| Surkhet | 5 | 544 | Pokharidanda CF | 566907 | 3161852 | 722 |
| Surkhet | 6 | 545 | Pokharidanda CF | 567046 | 3162743 | 771 |
| Surkhet | 1 | 546 | Sallaghari CF | 573530 | 3156672 | 1003 |
| Surkhet | 2 | 547 | Sallaghari CF | 573296 | 3156475 | 1009 |
| Surkhet | 3 | 548 | Sallaghari CF | 573623 | 3155651 | 1003 |
| Surkhet | 4 | 549 | Sallaghari CF | 573849 | 3155578 | 999 |
| Surkhet | 5 | 550 | Sallaghari CF | 574027 | 3155401 | 998 |
| Surkhet | 6 | 551 | Sallaghari CF | 574459 | 3155483 | 1003 |
| Surkhet | 7 | 552 | Sallaghari CF | 574734 | 3155266 | 1006 |
| Surkhet | 1 | 553 | Siddhapaila CF | 563034 | 3170612 | 1874 |
| Surkhet | 2 | 554 | Siddhapaila CF | 562628 | 3170168 | 1974 |
| Surkhet | 3 | 555 | Siddhapaila CF | 562905 | 3169709 | 1820 |
| Surkhet | 4 | 556 | Siddhapaila CF | 563321 | 3169126 | 1509 |
| Surkhet | 5 | 557 | Siddhapaila CF | 563134 | 3169477 | 1667 |
| Surkhet | 1 | 558 | Tilkakhal Mahila CF | 549691 | 3177074 | 668 |
| Surkhet | 2 | 559 | Tilkakhal Mahila CF | 541924 | 3177107 | 738 |
| Surkhet | 3 | 560 | Tilkakhal Mahila CF | 549304 | 3176807 | 597 |
| Surkhet | 4 | 561 | Tilkakhal Mahila CF | 549199 | 3176664 | 587 |
| Tanahun | 1 | 562 | Mandre Kalika | 238424 | 3100421 |  |
| Tanahun | 2 | 563 | Mandre Kalika | 239024 | 3099671 |  |
| Tanahun | 3 | 564 | Mandre Kalika | 239774 | 3100571 |  |
| Tanahun | 4 | 565 | Mandre Kalika | 239624 | 3101021 |  |
| Tanahun | 5 | 566 | Mandre Kalika | 238274 | 3102521 |  |
| Tanahun | 6 | 567 | Mandre Kalika | 237824 | 3101921 |  |
| Tanahun | 1 | 568 | Shiva Proposed CF | 247057 | 3106880 |  |
| Tanahun | 2 | 569 | Shiva Proposed CF | 246357 | 3106980 |  |
| Tanahun | 3 | 570 | Shiva Proposed CF | 246757 | 3106880 |  |
| Tanahun | 4 | 571 | Shiva Proposed CF | 246257 | 3106780 |  |
| Tanahun | 5 | 572 | Shiva Proposed CF | 246957 | 3107080 |  |
| Udayapur | 1 | 573 | Belaka | 495047 | 2962829 | 168 |
| Udayapur | 2 | 574 | Belaka | 495647 | 2962529 | 194 |
| Udayapur | 3 | 575 | Belaka | 495647 | 2962829 | 227 |
| Udayapur | 4 | 576 | Belaka | 495347 | 2963129 | 198 |
| Udayapur | 5 | 577 | Belaka | 495047 | 2963129 | 181 |
| Udayapur | 4 | 578 | Chireshor Mahadevi | 449747 | 2979029 | 590 |
| Udayapur | 3 | 579 | Chireshor Mahadevi | 449447 | 2978729 | 565 |
| Udayapur | 2 | 580 | Chireshor Mahadevi | 449147 | 2978429 | 569 |
| Udayapur | 1 | 581 | Chireshor Mahadevi | 449447 | 2978129 | 613 |
| Udayapur | 1 | 582 | Damauti 2, Bilaune | 459647 | 2959229 | 194 |
| Udayapur | 2 | 583 | Damauti 2, Bilaune | 459647 | 2959529 | 179 |
| Udayapur | 3 | 584 | Damauti 2, Bilaune | 459647 | 2960129 | 195 |
| Udayapur | 4 | 585 | Damauti 2, Bilaune | 459947 | 2959829 | 193 |
| Udayapur | 5 | 586 | Damauti 2, Bilaune | 459947 | 2959229 | 178 |
| Udayapur | 1 | 587 | Devdhar | 480947 | 2963729 | 186 |
| Udayapur | 2 | 588 | Devdhar | 481247 | 2964029 | 180 |
| Udayapur | 3 | 589 | Devdhar | 481247 | 2964329 | 216 |
| Udayapur | 4 | 590 | Devdhar | 480947 | 2964329 | 270 |
| Udayapur | 5 | 591 | Devdhar | 480647 | 2964329 | 230 |
| Udayapur | 1 | 592 | Jajarkhola | 465974 | 2957429 | 238 |
| Udayapur | 2 | 593 | Jajarkhola | 465647 | 2957129 | 220 |
| Udayapur | 3 | 594 | Jajarkhola | 465647 | 2956829 | 214 |
| Udayapur | 4 | 595 | Jajarkhola | 465947 | 2956829 | 222 |
| Udayapur | 5 | 596 | Jajarkhola | 466247 | 2956829 | 230 |
| Udayapur | 6 | 597 | Jajarkhola | 466547 | 2957429 | 236 |
| Udayapur | 7 | 598 | Jajarkhola | 466247 | 2957429 | 234 |
| Udayapur | 1 | 599 | Jodgidaha | 479255 | 2955547 | 220 |
| Udayapur | 2 | 600 | Jodgidaha | 479655 | 2955747 | 229 |
| Udayapur | 3 | 601 | Jodgidaha | 479855 | 2955747 | 237 |
| Udayapur | 4 | 602 | Jodgidaha | 480055 | 2955947 | 244 |
| Udayapur | 1 | 603 | Sadabahar | 435647 | 2977829 | 377 |
| Udayapur | 2 | 604 | Sadabahar | 439547 | 2979629 | 314 |
| Udayapur | 3 | 605 | Sadabahar | 435047 | 2978429 | 328 |
| Udayapur | 4 | 606 | Sadabahar | 434747 | 2978842 | 341 |
| Udayapur | 5 | 607 | Sadabahar | 435047 | 2978129 | 321 |
| Udayapur | 6 | 608 | Sadabahar | 435647 | 2980529 | 248 |
| Udayapur | 7 | 609 | Sadabahar | 435047 | 2973929 | 291 |
| Udayapur | 1 | 610 | Saptakoshi | 513966 | 2970739 | 313 |
| Udayapur | 2 | 611 | Saptakoshi | 513716 | 2970869 | 423 |
| Udayapur | 3 | 612 | Saptakoshi | 524341 | 2970869 | 1066 |
| Udayapur | 4 | 613 | Saptakoshi | 514051 | 2971129 | 369 |
| Udayapur | 5 | 614 | Saptakoshi | 514466 | 2971259 | 323 |
| Udayapur | 1 | 615 | Shree Nawaprabhat | 479759 | 2956678 | 217 |
| Udayapur | 2 | 616 | Shree Nawaprabhat | 479447 | 2956229 | 216 |
| Udayapur | 3 | 617 | Shree Nawaprabhat | 479147 | 2955929 | 205 |
| Udayapur | 4 | 618 | Shree Nawaprabhat | 479147 | 2956529 | 193 |
| Udayapur | 5 | 619 | Shree Nawaprabhat | 479147 | 2956829 | 195 |
| Udayapur | 6 | 620 | Shree Nawaprabhat | 479447 | 2957129 | 194 |
