# Supplementary material for: The effects of the Nepal community forestry program on biodiversity conservation and carbon storage
Source: PLoS One. 2018 Jun 26;13(6):e0199526. doi: 10.1371/journal.pone.0199526 (PMC6019746; doi:10.1371/journal.pone.0199526)
Supplement: S2 Table — (DOCX) [file pone.0199526.s002.docx]

# S3 Table. Standardized Mean Difference Before and After Matching by Covariate.

|  | **Overall forest** | | **Lower altitude** | | **Higher altitude** | | **Lower slope** | | **Higher slope** | | **Terai** | | **Hill** | | **Open canopy** | | **Closed canopy** | |
| --- | --- | --- | --- | --- | --- | --- | --- | --- | --- | --- | --- | --- | --- | --- | --- | --- | --- | --- |
| **Covariates** | **BM SDM** | **AM SDM** | **BM SDM** | **AM SDM** | **BM SDM** | **AM SDM** | **BM SDM** | **AM SDM** | **BM SDM** | **AM SDM** | **BM SDM** | **AM SDM** | **BM SDM** | **AM SDM** | **BM SDM** | **AM SDM** | **BM SDM** | **AM SDM** |
| Forest size | 0.31 | 0.12 | 0.52 | 0.28 |  |  | 0.48 | 0.21 | 0.49 | 0.20 | 0.52 | 0.16 |  |  |  |  | 0.37 | 0.19 |
| Forest per household |  |  |  |  | 0.04 | 0.08 |  |  |  |  |  |  | 0.31 | 0.26 | 0.14 | 0.15 |  |  |
| Forest users households | 0.01 | 0.10 | 0.10 | 0.14 |  |  | 0.18 | 0.25 | 0.34 | 0.07 | 0.17 | 0.20 |  |  |  |  | 0.03 | 0.14 |
| Travel time to nearest road | 0.17 | 0.25 |  |  | -0.08 | 0.13 | 0.28 | 0.12 |  |  | 0.12 | -0.24 |  |  | 0.20 | 0.10 | 0.16 | 0.16 |
| Travel time to district headquarter |  |  | 0.37 | 0.14 |  |  |  |  | -0.07 | 0.28 |  |  | 0.06 | -0.04 |  |  |  |  |
| Slope | 0.86 | 0.18 | 0.72 | 0.23 | -0.09 | 0.01 | 0.70 | 0.01 | 0.24 | 0.14 | 0.56 | 0.15 | -0.1 | -0.00 | 0.85 | -0.03 | 0.89 | 0.17 |
| Altitude | 0.82 | 0.23 | 0.85 | 0.30 | -0.01 | 0.01 | 0.63 | 0.20 | 0.35 | 0.25 | 0.68 | 0.15 | -0.23 | -0.18 | 0.88 | 0.16 | 0.77 | 0.11 |
| Moisture gradient | -0.41 | 0.04 | -0.55 | -0.06 | 0.5 | 0.12 | -0.53 | 0.16 | 0.24 | 0.12 | -0.38 | 0.02 | 0.24 | 0.03 | -0.23 | 0.24 | -0.56 | -0.03 |
| Broadleaved-conifer gradient | 0.54 | 0.19 |  |  | -0.23 | -0.02 |  |  | 0.15 | 0.11 |  |  | -0.21 | -0.06 | 0.51 | -0.03 | 0.56 | 0.25 |
| Sal | -0.42 | 0.05 | -0.14 | -0.13 |  |  | -0.43 | 0.00 | -0.09 | 0.11 | -0.20 | -0.04 | 0.22 | 0.21 | -0.39 | -0.05 | -0.43 | -0.02 |
| Soil erosion | -0.07 | 0.03 | 0.09 | 0.09 |  |  | -0.03 | 0.06 | -0.34 | 0.04 | -0.15 | 0.11 | -0.20 | 0.11 | 0.26 | 0.03 | -0.32 | 0.09 |
| NDVI 1990 | -0.32 | -0.09 | -0.25 | -0.18 | 0.14 | 0.04 | -0.27 | -0.03 | -0.14 | -0.12 | -0.12 | 0.03 | 0.11 | 0.04 | -0.37 | -0.09 | -0.27 | -0.14 |
| Community conserving forest (years) | 0.92 | 0.02 | 0.54 | 0.18 |  |  | 0.65 | 0.25 | 1.01 | 0.48 | 0.58 | 0.25 |  |  |  |  |  |  |
| Proportion of ancestral home | 0.60 | 0.11 | 0.27 | -0.11 | 0.65 | 0.12 | 0.30 | -0.04 | 0.15 | 0.08 | 0.32 | -0.08 | 0.01 | 0.08 | 0.76 | -0.10 | 0.48 | 0.15 |
| Proportion od ethnic population | 0.13 | 0.10 | 0.38 | -0.03 | -0.28 | -0.05 | 0.23 | -0.01 | -0.01 | 0.10 | 0.35 | 0.14 |  |  | 0.19 | 0.02 | 0.06 | 0.02 |
| Proportion of poor population | -0.05 | 0.06 | -0.01 | 0.05 | -0.39 | 0.22 |  |  | -0.02 | 0.23 | -0.57 | -0.11 | 0.09 | 0.04 | -0.25 | 0.02 | 0.11 | 0.23 |

Note: overall= overall forest of the country, lower altitude = <1000m, higher altitude = ≥1000m, lower slope = <15 degree, higher slope = ≥15 degree, open canopy=<50%, closed canopy= ≥50%, Terai= political districts of southern plain land, hill= political districts except southern plain land.
