# Supplementary material for: The effects of the Nepal community forestry program on biodiversity conservation and carbon storage
Source: PLoS One. 2018 Jun 26;13(6):e0199526. doi: 10.1371/journal.pone.0199526 (PMC6019746; doi:10.1371/journal.pone.0199526)
Supplement: S1 Text — (PDF) [file pone.0199526.s003.pdf]

Supplementary Information

This section includes the questionnaires used to collect data from the non-community forestry user groups (both Nepali and English versions), and the community and non-community forests (as we used, English version only). As the questions for the non-community forest user group data collection is adapted from the community forestry impact study conducted by the Ministry of Forest and Soil Conservation in 2010-2012 (MoFSC, 2013), the serial number of the questionnaires were kept to match the earlier study.

Reference

Ministry of Forest and Soil Conservation [MoFSC]. (2013). *Persistence and Change: Review of 30 Years of Community Forestry in Nepal.* Ministry of Forest and Soil Conservation, Nepal.

क्रम संख्याः..................

सामुदायिक वन र रेड

<u>समुह सर्वेक्षण प्रश्नावली -सरकारी वन (Non CFUG)</u>

<u>कोडिङ्ग नोट</u>

१. उत्तरदाताले 'जान्दिन' वा उत्तर 'काम नलाग्ने छ' भनेमा उत्तर खाली छोडिदिनुस् ।

२. उत्तरदाताले 'होइन' जुनसुकै कारणले भने पनि उत्तर खाली छोडिदिनुस् ।

३. उत्तर शुन्य भए, ० लेख्नुस् ।

४. 'काम नलाग्ने' वा 'होइन' उत्तरलाई ० नलेख्नुस् ।

५. 'हो/होइन' भन्ने प्रश्नमा 'हो' लाई १ र होइनलाई ० राखिदिनुस् ।

६. उत्तरदाताहरुलाई विकल्प दिँदा पहिलो विकल्पलाई सधैं १, दोस्रो विकल्पलाई २, तेस्रोलाई ३ एवं प्रकारले दिने ।

७. लाईकर्ट स्केलको हकमा कोडहरु (जस्तै ५, ४, ३ , २, १) प्रयोग गर्नुस् ।

यो प्रश्नावलीको जवाफहरु अनुसन्धान प्रयोजनका लागि मात्र प्रयोग गरिनेछ ।

यसबाट आएका जवाफहरुमा गोपनियता राखिनेछ ।

खण्ड : सामुदायिक वन उपभोक्ता समूहको परिचय

१_१ सामान्य जानकारी

१_१_१ जिल्लाः

१_१_२ रेञ्जपोष्टः

१_१_३ समूहको नामः

१_१_४ समूह रहेको गाविसहरू र वडा नं. (छुट्टाछुट्टै गाविसको नाम र वडा नं. उल्लेख गर्ने)ः

- ☐
- ☐
- ☐

१_१_५ वनको क्षेत्रफलः .... हेक्टर

क. प्राकृतिक वन क्षेत्र कति छ ? ....हेक्टर

ख. वृक्षारोपण भएको जग्गा कति छ ? ....हेक्टर

ग. अन्य जग्गा कति छ (खुलाउनुहोस्) ? ....हेक्टर

१_१_६ जम्मा घरधुरी संख्याः ....

दलित		ज्नजाति			ब्राम्हण/क्षेत्री		मुस्लिम १_१_१४	मधेशी पिछडावर्ग १_१_१५	अन्य १_१_१६	जम्मा
पहाडी १_१_७	मधेशी १_१_८	अन्य		नेवार १_१_११	पहाडी १_१_१२	मधेशी १_१_१३				
		पहाडी १_१_९	मधेशी १_१_१०							

<u>१।१।७ घरमुली सम्बन्धि जानकारी</u>

१।१।७।१ महिला घरमुली हुने घरधुरीको संख्याः ☐ विधुवा ☐ घरमुली वाहिर गएका घरधुरी ☐ अन्य

१।१।८ वन समुह गठन भएको मितिः

१।१।९ वनको किसिम ☐ चौडापाते ☐ कोणधारी ☐ मिश्रित

१।१।१० वन कुन किसिमको छ ? ☐ पूर्ण प्राकृतिक ☐ पूर्ण वृक्षारोपण ☐ मिश्रित

१।१।११ मुख्य प्रजातिहरूः वनका किसिमका आधारमा

१।१।१२ वनको अवस्था कस्तो छ ? ☐ कमसल ☐ मध्यम/ठिकै ☐ राम्रो

१।१।१२।१ (क) छत्र घनत्वः ☐ पातलो ☐ मध्यम/ठिकै ☐ बाक्लो

१।१।१२।२ (ख) वनको उमेर (Strata): ☐ विरूवा /लाथ्रा ☐ पोल/खावा/वल्लावल्ली ☐ रूख

१।१।१२।३ (ग) पुनर्उत्पादनको अवस्थाः ☐ कमसल ☐ मध्यम/ ठिकै ☐ राम्रो

१।१।१३ नजिकमा यो वन उपभोक्ता समूहका सदस्यले प्रयोग गर्न सक्ने अन्य कुनै वन छ कि छैन ?

☐ छ ☐ छैन

११।१४ नजिकको मूल सडक पैदल जान र फर्कन लाग्ने समयः ❑ २ घण्टा भन्दा कम ❑ २ घण्टा भन्दा बढी
❑ आधा दिन भन्दा कम ❑ आधा दिन भन्दा बढी

११।१५ सदरमुकामबाट वन जान लाग्ने समयः ❑ २ घण्टा भन्दा कम ❑ २ घण्टा भन्दा बढी
❑ आधा दिन भन्दा कम ❑ आधा दिन भन्दा बढी

१।३ सदस्यता सम्बन्धी विवरण

१।३।३ सुरूमा वन उपभोक्ता समूह गठन हुँदा सदस्य शुल्क लिइएको थियो ? ❑ थियो ❑ थिएन

१।३।४ यदि थियो भने कती शुल्क लिइएको थियो ? .... रूपैयां

१।३।५ नयाँ उपभोक्ता बन्न खोज्ने परिवारलाई सदस्यता शुल्क लिइन्छ ? ❑ लिइन्छ ❑ लिइदैन

१।३।६ यदि लिइन्छ भने, कति शुल्क लिइन्छ ? .... (फरक शुल्क भए तपशिल अनुसार छुट्याउनु होस)

छुट्टिभिन्न भएका .... रूपैयां नयाँ.............. रूपैयां अन्य..............रूपैयां

१।३।७ तपाईको वन उपभोक्ता समूहका सदस्यहरू अन्य सामुदायिक वनका पनि उपभोक्ता छन् ?

❑छन् ❑छैनन् छन् भने कुन समूहमा कति जना उपभोक्ता भएका छन् ?

❑

❑

१।५।१ के तपाईको वन, प्रस्तावित सामुदायिक वन हो ? ❑ हो ❑ होइन

	**अति सहमत (५)**	**सहमत (४)**	**मध्यम (३)**	**असहमत (२)**	**अति असहमत (१)**
आफुले प्रयोग गर्ने वनको सिमाना प्रष्ट रूपमा यकिन गर्न सकिन्छ भन्नेकुरामा तपाई कतिको सहमत हुनुहुन्छ ? (जुन हस्तान्तरण भैसेकेको सामुदायिक वन वा अन्य ठुला क्षेत्र ओगटेका वनबाट छुट्याउन सकिन्छ)					

१।५।२ कति घरधुरीले यस वनबाट फाइदा पाइरहेको छन् ? ....

१।५।३ फाइदा पाउनुको मापदण्डहरू के के हुन् ? (बहुउत्तर हुन सक्ने)

१। सदस्यता

२। वन क्षेत्र नजिक बसोबास

३। योगदान अनुसार

४। अन्य (खुलाउनुहोस्)

१।५।४ कति घर धुरी वन पैदावारमा आश्रित छन् ?.................................. त्यसमध्ये

१।५।५ कति उपभोक्ताहरू घरायसी आय आर्जन/व्यापारीक प्रयोजनको लागि निर्भर छन् ? ....

१।५।६ समुहको सदस्यता ऐच्छिक र अनिवार्य के छ ? ❑ ऐच्छिक ❑ अनिवार्य

	अति धेरै (५)	धेरै (४)	ठिकै (३)	कम (२)	अति कम (१)	छैन (०)
१।५।७ स्रोत र सम्पत्तिको आधारमा सदस्य घरधुरीहरू बीच कतिको भिन्नता छ भन्ने कुरामा तपाई कतिको सहमत हुनुहुन्छ ?						

	अति सहमत (५)	सहमत (४)	मध्यम (३)	असहमत (२)	अति असहमत (१)
१।५।८ वन समुहमा स्रोतको अधिकारिक प्रयोग गर्ने र नगर्ने प्रष्ट रूपमा चिनिन्छ भन्नेकुरामा तपाई कतिको सहमत हुनुहुन्छ ?					

१।५।९ वन व्यवस्थापनको दृष्टिकोणले सदस्यहरू बीच कतिको विश्वास छ ? ❑ छ ❑ छैन

१।५।१० के वन उपभोक्ताहरूले चाहेको खण्डमा नेतृत्व परिवर्तन गर्न सक्दछन् ?

१। सक्दैनन्

२। सक्छन् (नियमित साधारण सभाद्धारा)

३। सक्छन् (साधारण सभाद्धारा जुन चाहिएको बेला गर्न सकिन्छ)

४। अन्य खुलाउनुस्

१।५।११ वन उपभोक्ता सदस्यहरूले आफ्नो चासोको विषय तथा सरोकारहरू सम्बन्धित निकायमा कसरी प्रस्तुत गर्दछन् ?

१। औपचारिक निवेदन २। औपचारिक सुनुवाई ३। सामान्य वैठक
४। अनौपचारिक छलफल (सम्बन्धित निकायहरूसँग) ५। अन्य खुलाउनुहोस

१।५।१२ के निर्णय प्रक्रिया समुह स्थापना भए देखिनै उस्तै छ ? ❑ छ ❑ छैन

छैन भने के के फरक परेको छ ?

	अति सहमत (५)	सहमत (४)	मध्यम (३)	असहमत (२)	अति असहमत (१)
१।५।१३ समिति उपभोक्ताहरू प्रति जवाफदेहि हुन्छन् भन्ने कुरामा तपाई कतिको सहमत हुनुहुन्छ ?					
१।५।१४ समिति पनि उपभोक्ता हुन् भन्ने कुरामा तपाई कतिको सहमत हुनुहुन्छ ?					
१।५।१५ कुनै पनि सदस्य/उपभोक्ताको विश्वास, समुहको नीति कति मानेका छन् त्यसमा भर पर्छ भन्ने कुरामा तपाई कतिको सहमत हुनुहुन्छ ?					

१।५।१६ तपाईको समुहमा कुनै आन्तरिक मतभेद भएको छ ? ❑ छ ❑ छैन यदि छ भने ....

१।५।१७ द्वन्द निरूपण गर्ने कुनै माध्यम छ ? ❑ छ ❑ छैन

१।५।१७।१ यदि छन् भने कुन पद्धति लगाएर द्वन्द निरूपण गर्ने गरिन्छ ? (कुनै उदाहरण सहित उल्लेख गर्नुस्)

....

....

....

	अति सहमत (५)	सहमत (४)	मध्यम (३)	असहमत (२)	अति असहमत (१)
१।५।१८ उपभोक्ता र सम्बन्धित निकायसँग द्वन्द (उपभोक्ता बीच र उपभोक्ता र सरकारी निकायहरू बीच) निवारण गर्ने क्षमता छ भन्ने कुरामा तपाई कतिको सहमत हुनुहुन्छ भन्ने कुरामा तपाई कतिको सहमत हुनुहुन्छ ?					

१।५।१९ के तपाईहरूको अन्य छिमेकी समुहसँग विवाद छ ? ❑ छ ❑ छैन

	अति धेरै (५)	धेरै (४)	ठिकै (३)	थोरै (२)	अति थोरै (१)
१।५।१९।१ यदि छ भने कस्तो छ ?					

१।५।१९।२ कसरी यस्ता विवादहरू समाधान गरिन्छ ?

....

....

१।५।२० सरकारी निकायबाट समुहको गतिविधिमा हस्तक्षेप गरेको कुनै घटना छ ?❑ छ ❑ छैन

छ भने विवरण दिनुस् ....

....

....

	अति सहमत (५)	सहमत (४)	मध्यम (३)	असहमत (२)	अति असहमत (१)
१।५।२१ समुहका सदस्यहरूलाई आफ्नो संस्था आफै संचालन गर्न पाउने अधिकार लाई वाह्य सरकारी निकायबाट चुनौति दिइएको छैन भन्ने कुरामा कति सहमत हुनुहुन्छ ?					

१।५।२२ के सदस्यहरूले समुहमा कुनै समस्या पर्दा विशेष साधारण सभा बोलाउन सक्छन् ? ❑ सक्छन् ❑ सक्दैनन्

१।५।२२।१ यदि छन् भने गत बर्षमा त्यस्तो विशेष सभा बोलाइएको छ ? ❑ छ ❑ छैन

१।५।२२।२ यदि छैन् भने पछिल्लो ५ बर्षमा त्यस्तो सभा बोलाइएको छ ? ❑ छ ❑ छैन

१।५।२३ समुहले (वन समुहको सल्लाह अनुरूप) कुनै नीति नियम परिवर्तन गरेको छ ? ❑ छ ❑ छैन

१।५।२४ के तालिम र प्राविधिक सहायताका लागि बाहय संघ संस्थाहरूको संलग्नता छ ? ❑ छ ❑ छैन

१।५।२४।१ यदि छ भने त्यस्तो संघ संस्थाको संलग्न्ताबारे बताउनुस् ।

....

....

१।५।२५ के तालिम र प्राविधिक सहायताका लागि स्थानिय संघ संस्थाहरूको संलग्न्ता छ ? ❑ छ ❑ छैन

१।५।२५।१ यदि छ, भने त्यस्तो संस्थाहरूको संलग्नताबारे बताउनुस् ।

....

....

१।५।२६ के वन स्रोत व्यवस्थापनमा वाह्य संघ संस्थाको संलग्न्ता छ ? ❑ छ ❑ छैन

१।५।२६।१ यदि छ भने स्रोत ब्यवस्थापनपनमा संलग्न्ताबारे बताउनुस् ।

....

....

१।५।३० तपाईको समुदायमा अन्य कुनै वन समुह अथवा सामुदायिक वन समुहहरू छन् ? ❑ छन् ❑ छैनन्

१।५।३१ यदि छन् भने तिनीहरूले पनि उस्तै नीति, नियम र अनुगमनका तरिकाहरू लागु गर्दछन् ? ❑ छन् ❑ छैनन्

१।५।३२ यदि छैनन भने कसरी व्यवस्थापन भएको छ ?

....

....

<u>सामुदायिकको नीति नियम सम्बन्धि थप प्रश्नहरू</u>

(नोटः नीति र नियमहरू अनौपचारिक पनि हुन सक्छन त्यसैले जवाफ कर्तालाई (Respondent) लाई आफ्नो समुहमा भएको नीति र नियमहरू गहिरिएर सोच्न प्रेरित गर्नुहोस् ।)

१।६। के समुहको कुनै अनौपचारिक, औपचारिक, नीति नियम छन्, जसले स्रोतका पहुच, प्रयोग, व्यवस्थापन जस्ता कुराहर निर्धारण गर्दछ ?❑ छ ❑ छैन

यदि छ भने, तलको प्रश्नहरूको उत्तर दिनुस् ।

१।६।१ के त्यस्तो नीति, नियमहरू लिखित छन् ?❑ छ ❑ छैन

१।६।२ त्यस्तो नीति, नियमहरू बनाउन समुहले विशेष व्यक्ति अथवा समुह तोकिदिएको छ ?❑ छ ❑ छैन

	अति सहमत (५)	सहमत (४)	मध्यम (३)	असहमत (२)	अति असहमत (१)
१।६।३ वनको कार्ययोजना सम्बन्धित सबै व्यक्ति/ सदस्यलाई समुहमा समावेश गरिएको छ भन्ने कुरामा तपाई कतिको सहमत हुनुहुन्छ ?					

१।६।४ समुहले बनाएको नीति नियम कसले लागु गर्दछ ?

१। समुह आफैले

२। समुहले तोकेको वन हेरालुले

३। सरकारी कर्मचारीले

४। अन्य खुलाउनुहोस्

१।६।५ यदि वन हेरालुबाट हुन्छ भने समुहले वन संरक्षण गर्न त्यस्ता वन हेरालु कसरी चयन गर्दछन् ?

१। सभाद्वारा छानिन्छ

२। लौरे पालो

३। अन्य (खुलाउनुहोस्)

१।६।६ समुहले कसमाथि वन अपराध सम्बन्धि नीति नियमहरू लागु गर्दछन् ?

१। समुहभन्दा बाहिरको व्यीक्तहरूलाई

२। समुहको सदस्यलाई

३। दुवै

४। अन्य खुलाउनुस्

१।६।७ यी नीति नियमहरू कसरी लागु गरिन्छ ?

१। गस्ती गरेर २। अन्य खुलाउनुस्

१|६|७|१ यदि गस्ती हो भने, गस्ती गर्ने तरिका बताउनुस् ।

....

....

१|६|७|२ त्यस्ता गस्तीहरू कति समयमा गरिन्छ ?

१| दिनको दुइ पटक

२| दिनको एक पटक

३| हरेक दोस्रो दिन

४| साप्ताहिक

५| अन्य (खुलाउनुस्)

१|६|७|३ त्यस्ता गस्तीहरू कुन समयमा गरिन्छ ?

१| विहान र बेलुका

२| प्रत्येक विहान

३| प्रत्येक बेलुका

४| अन्य खुलाउनुस्

१|६|७|४ यदि गस्ती गर्ने काम सदस्यहरूबाट हो भने तिनीहरूलाई कसरी प्रत्साहित गरिन्छ ?

....

....

१|६|८ के समुहले स्रोतको पहुँच, प्रयोग, व्यवस्थापनसँग सम्बन्धित नीति नियम आफुले चाहेको बेलामा परिवर्तन गर्न सक्छन् ?

१| सक्छन   २| सक्दैनन्

१|६||८|१ यदि सक्छन भने, कुन प्रक्रियाबाट गर्छन् ?

....

....

१|६|९ तपाईको विचारमा के सदस्यहरूले वन पैदावार प्रयोग गर्दा नीति नियमको पालना गर्दछन् ?

१| गर्दैनन्   २| कहिलेकाँही   ३| आधा जस्तो गर्दछन्   ४| प्राय जस्तो गर्दछन्   ५| सधै गर्दछन्

१|६|१० के सरकारी कर्मचारी कुनै तरिकाले समुहमा नीति नियम पालन नगर्ने सदस्यहरूलाई सहयोग गर्दछन् ?

❑ गर्छन   ❑ गर्दैनन्

१|६|११ के तपाईको समुहमा जरिवाना गर्ने प्रवधान छ ? ❑ छ   ❑ छैन

१|६|११|१ यदि छ भने, जरिवानाको निर्णय कसले गर्दछ ?

१| वन हेरालुले   २| समुहमा छलफल गरेर   ३| कार्यसमितिमा छलफल गरेर

४| कुनै अन्य समुह/समुदाय/वैधानिक निकायहरूमा छलफल गरेर   ५| अन्य (खुलाउनुस्)

१|६|११|२ यदि हो भने, जरिवाना के के तिर्नुपर्छ ? ❑ नगद ❑ जिन्सी   ❑ अन्य

१|६|११|२|१| जरिवाना कस्ले तिराउँछ ? ....

१| समूहको वनपाले जसले नियम तोड्नेलाई पकडिन्छ   २| समितिका सदस्यहरूले

३| गा.वि.स.का पदाधिकारीहरूले   ४| अन्य बयान गर्नुस ....

१।६।११।२।२। यदि हो भने यो जरिवानाको प्रयोग के मा गरिन्छ ?...

१। समुहको कोष २। स्थानिय निकायको राजस्वको रूपमा

३। वन विभाग/ वन कार्यालयको राजस्वको रूपमा ४। अन्य बयान गर्नुस्...

१।६।११।२।३ दण्डित गरिएका उपभोक्ताहरूले कतिको जरिवाना पालना गर्दछ ?

१। कोहीले पनि पालन गर्देनन् २। कसैकसैले पालना गर्दछन्

३। आधा जस्तोले पालना गर्दछन् ४। धेरै जस्तोले पालना गर्दछन् ५। सधैले पालना गर्दछन्

१।६।११।२।४ यदि उपभोक्ताले जरिवाना नतिरे सम्बन्धित समुहले के गर्ने गर्दछ ?

१। व्यक्तिगत रूपमा सम्झाएर जरिवाना पालना गर्न लगाइन्छ

२। सार्वजनिक गरेर जरिवाना पालना गर्न लगाइन्छ

३। जेल चलान गरिन्छ

४। उपभोक्ता अदालतमा लगिन्छ

५। अन्य खुलाउनुस्

	अति सहमत (५)	सहमत (४)	मध्यम (३)	असहमत (२)	अति असहमत (१)
१।६।१२ साधारण तथा अपराधको प्रकृति हेरी सानो अपराधलाई सानो र ठुलोलाई ठूलो सजाय गरिन्छ।					

खण्ड : सेवा र सम्पत्तिमा पहुँच (वन स्रोत सर्वेक्षण र सम्बर्द्धन सम्बन्धी)

२।०।१ के तपाईको वनको बृक्षारोपन योजना छ ? ❑ छ ❑छैन

२।०।२ के तपाईको समुहले सक्रिय भएर वन संरक्षण गरेको छ ? ❑ छ ❑छैन

१_२ वन कार्ययोजनाबाट संकलन गर्नुपर्ने जानकारीहरू

के तपाईको वनको कार्ययोजना छ ? ❑ छ ❑छैन

के तपाईहरू सक्रिय रूपमा वन संरक्षणमा सहभागी हुनुहुन्छ ? ❑ छ ❑छैन

	घट्दो (१)	कुनै परिवर्तन नभएको (२)	बढ्दो (३)
२।१।१ तपाईको विचारमा पछिल्लो ५ बर्षमा वनको अवस्था कस्तो परिवर्तन भएको छ ? (BIO MASS)			
२।१।२ तपाईको विचारमा पछिल्लो ५ बर्षमा वनको वृक्ष विविधतामा परिवर्तन भएको छ ?(TREE DIVERSITY)			
२।१।३ तपाईको विचारमा सामुदायिक वनले उपभोक्ताको जीविकोपार्जनमा सहयोग पुराएको छ ?			

१_२_६ वन संरक्षण भएपछि वन पैदावारको उपलब्धतामा आएको परिवर्तन

क्र. सं.	वन पैदावारको किसिम	अवस्था			विद्यमान अवस्था रहनुको कारण
		बढेको	घटेको	यथावत	
१	काठ				
२	दाउरा				
३	घांस				
४	पत्कर				
५	स्याउला/सोत्तर				
६	कृषि औजार				
७	जडिबुटी/NTFPs				
८	अन्य				

१_२_७ भएदेखि आर्थिक वर्ष सम्मको स्थिति हेर्दा सामुदायिक वन व्यवस्थापनले वनको अवस्थामा के कस्तो परिवर्तन ल्याएको देखिएको छ?

क्र.सं.	परिवर्तनका क्षेत्र	बढेको	घटेको	उस्तै	कारण	कैफियत
१	रूखले ढाकेको क्षेत्रफल					
२	रूखको घनत्व					
३	रूखको प्रजातीहरू					
४	वनको उत्पादकत्व					
५	पुनर्उत्पादन					
६	जैविक विविधता					
७	अन्य					

खण्ड : सेवा र सम्पत्तिमा पहुँच (समूहको आर्थिक पक्ष)

१_३ आम्दानी सम्बन्धी जानकारी

समूहको आम्दानी बहीखाता हेरी तलको तालिकामा दिएको आम्दानीको शीर्षक र स्रोत अनुसार रकम रू. मा भर्नुपर्ने

क्र.सं.	आम्दानीको शीर्षक	जम्मा आम्दानी रकम रू. मा		
		आ.व. २०६७/६८	आ.व. २०६८/६९	आ.व. ०६९/७०
१	अघिल्लो वर्षको बाँकी (अ.ल्या.)			
१.१	वैंक मौज्दात			
१.२	नगद मौज्दात/पेश्की/बेरूजु			
१.३	आय आर्जनका लागि ऋण लगानी			
१.४	अन्य ऋण लगानी			
	**जम्मा**			
२	काठ बिक्री			
३	दाउरा बिक्री			
४	खाँवा (पोल) बिक्री			
५	कृषि औजार बिक्री			
६	घांस र स्याउला बिक्री			
७	गैर काष्ठ वन पैदावार बिक्री			
८	अनुदान (स्थानीय निकाय)			
९	अनुदान अन्य.....................			
१०	ऋण (समूहले लिएको)			
११	जरिवाना			
१२	ब्याज			
१३	पेश्की बेरूजु			
१४	अनुसन्धान गर्न दिए बापत सेवा शुल्क			
१५	सदस्यता/वार्षिक/नविकरण शुल्क			
१६	प्रवेश शुल्क			
१७	कार्यक्रम सहयोग			
१८	अन्य			
	**जम्मा**			
	**कूल जम्मा**			

१_४ खर्च सम्वन्धी जानकारी

समूहको खर्च सम्बन्धी बहीखाता हेरी तलको तालिकामा खर्चको शीर्षक र स्रोतको आधारमा खर्चको विवरण भर्नुपर्ने

क्र.सं.	खर्चको शिर्षक	जम्मा खर्च रकम रू. मा		
		आ.व. २०६७/६८	आ.व. २०६८/६९	आ.व. ०६९/७०
	वन विकास कार्यकम			
	सामुदायिक विकास			
	सामुदायिक सेवा			
	उद्यम कार्यकम			
	आय आर्जन			
	तालिम, गोष्ठी तथा अध्ययन भ्रमण			
	प्रशासनिक खर्च			
	ब्याज, चन्दा, अनुदान			
	यो वर्षको वांकी मौज्दात			
	कुल जम्मा			

१_५ वन पैदावार उत्पादन तथा बिक्री वितरण सम्वन्धी जानकारी (गत आर्थिक वर्षको २०६८/६९)

समूहको उत्पादन तथा बिक्री वितरण सम्बन्धी बहीखाता हेरी वन पैदावार सम्बन्धी जानकारी भर्नुपर्ने

क्र.सं.	वन पैदावार को किसिम	इकाई	कुल उत्पादन परिमाण	सामाजिक समूह (१.दलित, २.जनजाती (अन्य), ३. जनजाती (नेवार), ४.ब्राम्हण/क्षेत्री, ५. मुस्लिम, ६. मधेशी पिछडा वर्ग, ७. अन्य)	आन्तरिक बिक्री वितरण								बाह्य बिक्री वितरण	
					निःशुल्क		सःशुल्क							
							छुट मूल्य			पूरा मूल्य				
					घरधुरी	परिमाण	घरधुरी	परिमाण	रकम रू.	घरधुरी	परिमाण	रकम रू.	परिमाण	आम्दानी
१	काठ	क्यू. फिट												
२	दाउरा	के.जी												
३	खावा (पोल)	गोटा												
४	कृषि औजार	गोटा												

क्र.सं.	वन पैदावार को किसिम	इकाई	कुल उत्पादन परिमाण	सामाजिक समूह (१.दलित, २.जनजाती (अन्य), ३. जनजाती (नेवार), ४.ब्राम्हण/क्षेत्री, ५. मुस्लिम, ६. मधेशी पिछडा वर्ग, ७. अन्य)	आन्तरिक बिक्री वितरण										बाह्य बिक्री वितरण	
					निःशुल्क		सःशुल्क									
							छुट मूल्य			पूरा मूल्य						
					घरधुरी	परिमाण	घरधुरी	परिमाण	रकम रू.	घरधुरी	परिमाण	रकम रू.			परिमाण	आम्दानी
५	भुईघास (Forage)	के.जी														
६	डालेघास (Fodder)	के.जी														
७	पतकर (Leaf litter)	के.जी														

क्र.सं.	वन पैदावार को किसिम	इकाई	कुल उत्पादन परिमाण	सामाजिक समूह (१.दलित, २.जनजाती (अन्य), ३. जनजाती (नेवार), ४.ब्राम्हण/क्षेत्री, ५. मुस्लिम, ६. मधेशी पिछडा वर्ग, ७. अन्य)	आन्तरिक बिक्री वितरण								बाह्य बिक्री वितरण	
					निःशुल्क		सःशुल्क							
							छुट मूल्य			पूरा मूल्य				
					घरधुरी	परिमाण	घरधुरी	परिमाण	रकम रू.	घरधुरी	परिमाण	रकम रू.	परिमाण	आम्दानी
८	गैऱ्हकाष्ठ	के.जी												
९	अन्य													

	एकदमै सहमत (५)	सहमत(४)	मध्यम(३)	असहमत(२)	एकदमै असहमत(१)
३।७।२ वन पैदावार कसलाई कति दिने भन्ने नियम सदस्यको नगद तथा जिन्सी योगदानले निर्धारण गर्छ भन्ने कुरामा तपाई कतिको सहमत हुनुहुन्छ ?					
३।७।३ धेरै वन पैदावार प्रयोग गर्ने सदस्यले धेरै पैसा तिर्नु पर्छ भन्ने कुरामा तपाई कतिको सहमत हुनुहुन्छ ?					
३।७।४ खर्च र फाइदा सम्बन्धि नीतिहरू स्थानिय अवस्था अनुसार निर्धारण गरिन्छ भन्ने कुरामा तपाई कतिको सहमत हुनुहुन्छ ?					
३।७।५ स्रोतमा पहुँच हुने निर्णय न्यायोचित छ भन्ने कुरामा तपाई कतिको सहमत हुनुहुन्छ ?					
३।७।६ फाइदाको न्यायोचित वितरण भएको छ भन्ने कुरामा तपाई कतिको सहमत हुनुहुन्छ ?					

३।७।७ कुन तरिका प्रयोग गरेर वन पैदावार वितरण गर्नु हुन्छ ?

१। समुहमा संकलन गर्ने र बराबर वितरण गर्ने

२। व्यक्तिगत रूपमा संकलन तथा प्रयोग गर्ने

३। समुहमा संकलन गर्ने र निर्धारित तौरतरिका प्रयोग गरि वितरण गर्ने

४। अन्य (खुलाउनुहोस्)

३।७।८ कुनै समयमा वन पैदावार वितरण गर्ने तरिकामा परिवर्तन भएको छ ? ❑ छ ❑ छैन

३।७।८।१ यदि छ भने कारण के हो ?

....

....

३।७।९ वन पैदावार लाभ वितरणमा कुनै समस्या तथा उजुरी परेको छ ? ❑ छ ❑ छैन

३।७।९।१ यदि छ भने वन पैदावार लाभ वितरणका समस्याहरू के के हुन् ?

....

....

३।७।१० सदस्यबाट अपेक्षित योगदानहरू के के हुन ?

१। श्रम दान

२। आर्थिक सहयोग

३। अन्य कुनै (खुलाउनुहोस्)

४। केही पनि छैन

३।७।११ तपाईको समुहको अन्य कुनै वनसँग सम्बन्धित/समन्वय संस्थाहरूको सम्बन्ध कस्तो छ ?

१। समन्वय छैन

२। समन्वय छ तर स्वतन्त्र छौ

३। समन्वय गरेर निति र निर्माण निर्धारण गर्छौं

खण्ड : क्षमता विकास तथा सशक्तिकरण

४।१।१० समितिको बैठक र साधारण सभा बोलाउने र निर्णय गर्ने प्रक्रिया के हो ?

समुह/संरक्षण समितिका कामहरू	कसले गर्ने गरेको छ ?	साधारण सभाका कामहरू	कसले गर्ने गरेको छ ?
मिति र स्थान तोक्ने		मिति र स्थान तोक्ने	
विषयवस्तु तयारी/प्रस्ताव ल्याउने		विषयवस्तु तयारी/प्रस्ताव ल्याउने	
उपभोक्ता, समिति पदाधिकारीलाई खबर गर्ने		उपभोक्ता, समिति पदाधिकारीलाई खवर गर्ने	
निर्णय गर्ने		निर्णय गर्ने	

४।३ सार्वजनिक लेखापरीक्षण र सार्वजनिक सुनुवाई

४।३।१ सार्वजनिक लेखापरीक्षण गरेको छ छैन ? ❑ छ ❑ छैन

४।३।२ सार्वजनिक सुनुवाई गर्ने गरेको छ छैन ? ❑ छ ❑ छैन

४।५ वार्षिक प्रतिवेदन तथा लेखापरीक्षण

४।५।१ लेखापरीक्षण सम्पन्न गरेको छ छैन ? ❑ छ ❑ छैन छैन भने किन ....

खण्ड : सामाजिक समावेशीकरणः नीति, सामाजिक मूल्य, मान्यता र व्यवहारमा आवश्यक परिवर्तन

४।६ विधान र कार्ययोजना

४।६_१ समूहहरूको विधान र वन कार्ययोजनामा विपन्न तथा वन्चित वर्गका लागि के कस्ता किसिमका प्रावधानहरू राखिएका छन् । (छन् भने मात्रै सोध्नुस्)

क्र.सं.	विधान वा कार्ययोजनामा भएका प्रावधानहरू	स्थिति
१	वन पैदावारको विकी वितरण विपन्न तथा वन्चित घरधुरीहरूलाई निःशुल्क वा सहुलियत दरमा दिने प्रावधान	❑ छ ❑ छैन
२	महिला, विपन्न तथा वन्चित घरधुरीहरूलाई फाईदा पुग्ने गरी कोषको परिचालन सम्बन्धी प्रावधान	
	क) स्वास्थ्यमा	❑ छ ❑ छैन
	ख) शिक्षामा	❑ छ ❑ छैन
	ग) आयआर्जन र उद्यम	❑ छ ❑ छैन
	घ) अन्य (खुलाउने)	❑ छ ❑ छैन
३	महिला, विपन्न तथा वन्चित घरधुरीहरूलाई रोजगारी दिने सम्बन्धी प्रावधान	
	क) दैनिक ज्यालादारी	❑ छ ❑ छैन
	ख) नोकरी	❑ छ ❑ छैन
४	महिला, विपन्न तथा वन्चित घरधुरीहरूलाई बैठक, तालिम र अन्य विकास निर्माणका काममा सहभागी हुँदा प्रोत्साहन स्वरूप सहयोग गर्ने प्रावधान	❑ छ ❑ छैन
५	महिला, विपन्न तथा वन्चित घरधुरीहरूलाई तालिम, गोष्ठी तथा भ्रमणमा प्राथमिकता दिने प्रावधान	❑ छ ❑ छैन
६	महिला, विपन्न तथा वन्चित घरधुरीहरूलाई कार्यसमितिका मुख्य पदहरूमा प्रतिनिधित्व गराउने प्रावधान	❑ छ ❑ छैन
७	महिला, विपन्न वर्गलाई आयआर्जन गर्न सामुदायिक वनको जग्गा छुट्याउने प्रावधान	❑ छ ❑ छैन

४।६_२ उपभोक्ता समिति र समूहले गर्ने गरेका निर्णयहरू

कार्यक्रम	निर्णय कस्ले गर्छ?		
	समिति	समूह सदस्य	दुवै
संरक्षणको लागी वन क्षेत्र निर्धारण			
सदुपयोगको नीति नियम वनाउने			
संरक्षण तरिका, पहिचान तथा निर्धारण			
दण्ड जरिवाना कस्तो वनाउने वारे निर्णय			
दण्ड जरिवाना कार्यान्वियन गराउने			
वन पैदावार तथा फाईदा बाँड्ने बारेमा नियम बनाउने			
वन व्यवस्थापनको बारेमा योजना वनाउने			
रूख बिरूवाको छनौट			
नर्सरी व्यवस्थापन			
वृक्षारोपण			
समूहको आम्दानीको व्यवस्थापन			
तालिम भ्रमण वारेको निर्णय			
आयमूलक कार्यक्रमको निर्धारण			
सामुदायिक विकासका कार्यक्रमको निर्धारण			
अन्य कार्यक्रम (लेख्नुहोस)			

४।६_३ दाउरा घाँस वितरण गर्ने तरिका कसरी निर्धारण गरिएको हो ?

- ❑ अन्य सामुदायिक वनको सिकाईबाट गरेको
- ❑ वन प्राविधिकको सल्लाह अनुसार
- ❑ समूह आफैले
- ❑ समिति आफैले

५।२।२२ सरकारी वन प्राविधिकबाट प्राप्त सेवालाई कसरी मूल्यांकन गर्नुहुन्छ ?

- ❑ अति उत्तम
- ❑ उत्तम
- ❑ ठिकै
- ❑ खराब

५।२।२३ उपर्युक्त आधारमा (अति उत्तम, उत्तम, ठिकै, खराब) मूल्यांकन गर्नुको कारण के हो ?

- ❑ विना कुनै झन्झट सेवा प्रदान गरेको
- ❑ विना कुनै झन्झट सेवा प्रदान नगरेको
- ❑ चाहिएको सूचना दिएको
- ❑ चाहिएको सूचना नदिएको
- ❑ अन्य (खुलाउने)

५।२।२४ सुशासन (पारदर्शिता, जवाफदेहिता, समावेशी, सहभागितामूलक, र विधिको शासन) प्रवर्द्धन गर्ने संयन्त्र र प्रक्रियाहरू के के हुन् ?

- ❑ सार्वजनिक परीक्षण
- ❑ सार्वजनिक सुनुवाई
- ❑ केहि पनि गरेको छैन
- ❑ अन्य (खुलाउने)

५।२।२५ सुशासन प्रवर्द्धनका लागि समुह बाट अपनाइएको प्रक्रिया के के छन् ?

- ❑
- ❑
- ❑

५।२।२६ बजेट निर्माण प्रक्रियामा लैंगिक र सामाजिक समावेशीकरणका मुद्दालाई सम्बोधन गर्ने केही प्रावधान छ? ❑ छ

५।२।२६।१ यदि छ भने खुलाउनुहोस्ः

- ❑
- ❑
- ❑

५।२।२९नीति र रणनीति निर्माण प्रक्रियामा गरीब तथा वञ्चित वर्गलाई संलग्न गराउने प्रावधान छ ? ❑ छ

५।२।३० यदि छ भने खुलाउनुहोस् ।

- ❑
- ❑

५।२।३१ वार्षिक कार्यक्रम तयार गर्ने प्रक्रिया कस्तो छ ?

- ❑ आमसभाबाट तयार गर्ने
- ❑ कार्यसमितिले तयार गरी लागू गर्ने
- ❑ रेञ्जपोष्टले तयार गर्ने
- ❑ समितिले मस्यौदा तयार पारी आम भेलाबाट पारित गर्ने
- ❑ अन्य

५।२।३२ समूहले राखेकोको कर्मचारी विवरण

कर्मचारीको नाम, थर	पद	दलित				जनजाति						ब्राम्हण/क्षेत्री				मधेशी पिछडा वर्ग		मुस्लिम		अन्य		कैफियत
		पहाडी		मधेशी		अन्य				नेवार		पहाडी		मधेशी								
						पहाडी		म्धेशी														
		म	पु	म	पु	म	पु	म	पु	म	पु	म	पु	म	पु	म	पु	म	पु	म	पु	

५।२।३६ पछिल्लो दुई वर्षमा बसेको कार्यसमितिको बैठकको माइन्यूट हेर्दा जम्मा कति पटक बैठक बसेको देखिन्छ ?
.... पटक

५।४ नियमको उल्लंघन र सजाय दिइएको विवरण

५।४।१ समय सगसगै वन पैदावार सम्बन्धी अपराध (चोरी) बढेको वा घटेको कस्तो छ ?

❑ बढेको ❑ घटेको ❑ उस्तै

५।४।२ यी अपराधहरू प्रायःजसो पुरुष वा महिला कस्ले गर्छन र किन ?

❑ पुरूषले ❑ महिलाले ❑ दुवै

५।४।३ अपराधहरू प्रायः आफ्नै गाउँबाट हुन्छ कि बाहिरबाट ?

❑ गाउँबाट ❑ बाहिरबाट

५।४।४ कुन चाहि वन पैदावार प्रायः चोरी हुन्छ ?

❑ दाउरा ❑ घाँस ❑ साना काठ

❑ ठूला काठ ❑ जडिबुटि

५।४।८ वार्षिक स्वीकार्य काठ कटानीको लागि तोकिएको परिमाण कति छ ? ....

५।४।९ वार्षिक स्वीकार्य कटान भन्दा बढी (काठ) कटान भएका छन्/छैनन् ? ❑ छ ❑ छैन

५।४।१० के बार्षिक स्वीकार्य ...... भन्दा बढि काठ निकाल्ने निर्णय भएको छ ? ❑ छ ❑ छैन

५।४।११ वार्षिक स्वीकार्य कटान भन्दा बढी (काठ)कटान भएको भए सो को कारणहरू उल्लेख गर्नुहोस् ।

❑

❑

५।४।१२ कस्तो खालको रूखहरू कटान भएको छ ?

❑ ढलापडा ❑ सुकेका र टुप्पो भाचिएका ❑ बढी परिपक्व

❑ कांचो ❑ रोग/किरा लागेका ❑ अन्य

५।४।१८ वन समूहले नै वनको व्यवस्थापन गर्दाको अनुभवको आधारमा वन कार्यक्रमका सकारात्मक र अप्ठ्यारा पक्षहरू के के हुन्?

सकारात्मक पक्षहरू	अप्ठ्यारा तथा थप चुनौतिहरू

५।४।१९ महिला, गरीब र वञ्चितीमा परेकाका मुद्दालाई सम्बोधन, सुशासनको अभिवृद्धि, र वनको गुणस्तर सुधार बारे समूहमा भएका राम्रा कामहरू के के हुन्?

महिला, गरीब र वञ्चितिमा परेका मुद्दाको सम्बोधन	सुशासनको अभिवृद्धि	वनको गुणस्तर सुधार

५।४।२० महिला, गरीब र वञ्चितिमा परेकाका मुद्दालाई सम्बोधन, सुशासनको अभिवृद्धि, र वनको गुणस्तर सुधार बारे उपभोक्ता समूहमा सुधार गर्नुपर्ने कुराहरू के के हुन्?

महिला, गरीब र वञ्चितिमा परेका मुद्दाको सम्बोधन	सुशासनको अभिवृद्धि	वनको गुणस्तर सुधार

आफुले अनुभव गर्नु भएको टिप्पणी तथा सुझावहरू भए लेख्नुहोस् ।

....

....

....

....

आन्तरिक प्रयोजनको लागि

सूचना संकलन गर्दा छलफल गरिएका समूह सदस्यहरू

क्र.सं.	नाम	पद	टेलिफोन नम्बर	
			घरको	मोवाइल नम्बर

सूचना संकलन गर्नेको नामः
पदः
मितिः
हस्ताक्षरः

रूजु गर्नेको नामः
पदः
मितिः
हस्ताक्षरः

Serial no. ....

Community Forestry and REDD+

GROUP SURVEY QUESTIONNAIRE (NON-CFUG)

Coding Notes

1. FOR ANY QUESTION WHERE THE RESPONDENT'S RESPONSE IS "NOT APPLICABLE" OR "I DON'T KNOW," ETC, PLEASE LEAVE BLANK
2. IF NO ANSWER GIVEN FOR WHATEVER REASON, PLEASE LEAVE BLANK
3. IF RESPONSE IS THE NUMBER ZERO, PLEASE CODE AS 0.
4. DO NOT CODE NO RESPONSE OR NOT APPLICABLE AS ZERO
5. FOR "YES" AND "NO" QUESTIONS, "YES" IS CODED AS 1 AND "NO" AS 0
6. IF OPTIONS ARE GIVEN FROM WHICH RESPONDENTS CHOOSE, THE FIRST OPTION IS ALWAYS 1, SECOND IS 2, THIRD IS 3, ETC.
7. FOR LIKERT SCALE MEASURES, PLEASE USE THE CODES GIVEN (e.g. 5, 4, 3, 2, 1)

Section 1: A Brief Profile of the FUG

1.1 General information

1.1.1 District:

1.1.2 Rangepost:

1.1.3FUG/(Forest Protection Committee) FPC Name:

1.1.4 FUG/FPCcoverage (list all the VDCs with ward number covered by FUG/FPC):

❑ ❑ ❑

❑ ❑ ❑

1.1.5 Forest (area: …….hectares

(a) Natural forest area ……… hectares

(b) Plantation area ………… hectares

(c) Other area ………… hectares

1.1.6Total number of households:

Dalit		Janajati			Brahman/Chhetri		Muslim 1.1.14	Madhesi Class 1.1.15	Others 1.1.16	Total
Hill 1.1.7	Madhesi 1.1.8	Hill 1.1.9	Madhesi 1.1.10	Newar 1.1.11	Hill 1.1.12	Madhesi 1.1.13				

1.1.7 Household head information

1.1.7.1 Number of women-headed HH:

1.1.7.2 ❑Numberdue to death of male ……..
1.1.7.3❑Number due to male out migration….

1.1.8 FUG/FPC formation date (if applicable):

1.1.9 Type of forest (forest type covering more than fifty percent area): ❑ Broadleaved ❑ Coniferous ❑ Mixed

1.1.10 Category of forest: ❑Completely natural ❑Completely planted ❑Mixed

1.1.11Major species:

1.1.12Condition of forest: ❑Bad ❑ Moderate ❑ Good

1.1.12.1 Crown Density ❑Thin ❑Medium ❑Dense

1.1.12.2 Age/Strata of forest ❑Sapling ❑ Pole ❑ Tree ❑ Mixed type

1.1.12.3 Regeneration status ❑Bad ❑Moderate ❑Good

1.1.13Is there any open forest nearby that FUG/FPC members can use: ❑ Yes ❑No

1.1.14 Walking distance of FUG/FPCfrom nearest roadhead (two-way): ❑Less than 2 hr ❑More than 2 hr ❑Less than half day ❑More than half day

1.1.15Distance from FUG/FPC to district HQ: ❑Less than 2 hr ❑More than 2 hr ❑Less than half day ❑More than half day

1.3 Membership

1.3.1 Was there any membership fee when FUG/FPC was formed? ❑ Yes ❑ No

1.3.2 If yes, how much (in NRs.)? ....

1.3.3 Currently, is there any membership fee for new members? ❑ Yes ❑ No

1.3.4 If yes, how much (in NRs.)?

1.3.4.1 …….for separated households,

1.3.4.2 ………..for new migrated households,

1.3.4.3 for others…………

1.3.5 Are there any memberswho are also members of other FUGs? ❑ Yes ❑ No

1.3.5.1 No. of repeated FUGs:

1.3.5.2. No of HHs:

1.5 ADDITIONAL BASIC INFORMATION

1.5.0 Is your community forest a proposed CFUG? ❑ Yes ❑ No

	Strongly agree(5)	Agree(4)	Neutral (3)	Disagree (2)	Strongly disagree (1)
1.5.1. The forest has clear boundaries that clearly separate it from the larger biophysical environment and that of other adjacent community forests.					

1.5.2. What is the number of households (in the community of users) that benefit from the resource? ____________

1.5.3. What are the criteria to be beneficiaries? (Multiple answers possible)
(1) Membership (2) Residence in the village (3) Input contribution (4) Others (specify) ______________________

1.5.4. What portionof households (in the community of users) depends significantly on this resource/forest for their own consumption? ________

1.5.5. What portion of (in the community of users/ beneficiaries) depends significantly on this resource/forest for their family income arising from commercial activities? _________

1.5.6. Can members (in the community of users/ beneficiaries) enter and exit voluntarily? ❑ Yes ❑ No

	Very highly (5)	Highly(4)	Averagely (3)	Less (2)	Very Less (1)	None (0)
1.5.7. How different are the members (in the community of users) in terms of wealth and assets?						

	Strongly agree(5)	Agree(4)	Neutral (3)	Disagree (2)	Strongly disagree (1)
1.5.8. The community forest has clear boundaries between legitimate users and nonusers and nonusers are effectively excluded.					

1.5.9. Do you believe that on matters related to natural resource management people/members in the community are trustworthy? ❑ Yes ❑ No

1.5.10. Can forest users remove the leaders (decision makers) when necessary?

(1) No (2) Yes, in elections that are held regularly (3) Yes, in elections that are held irregularly

(4) Yes, in arenas where criticisms are voiced (5) Others (describe) ________________

1.5.11. Indicate how members of the community of users (user group) express their needs and concerns about the forest (or any complaints) to officials of the community.

(1) Formal petition (2) Formal hearings (3) General meetings (4) Informal exchanges with officials

(5) Others (describe)____

1.5.12. Has the decision process always been the same since the establishment of the forest user group/FPC?

❑ Yes ❑ No

	Strongly agree(5)	Agree(4)	Neutral (3)	Disagree (2)	Strongly disagree (1)
1.5.13. Monitors who actively audit physical conditions and user behavior are accountable to the users.					
1.5.14. Monitors are the users themselves.					
1.5.15. Trustworthiness of people/members in the community is related with enforcement of rules.					

1.5.16. Have there been any serious internal conflicts within the forest user group/FPC?

❑ Yes ❑ No

1.5.17. Are there any mechanisms available for resolving internal conflicts?

❑ Yes ❑ No

1.5.17.1. If yes, how do internal conflicts in the community get resolved or what is the conflict resolution mechanism available? Could you please also give example?

	Strongly agree(5)	Agree(4)	Neutral (3)	Disagree (2)	Strongly disagree (1)
1.5.18. Users and their officials have rapid access to low-cost local arenas to resolve conflict between users or between users and officials.					

1.5.19. Were there conflicts with neighboring communities in the past such due to poachers or intruders?

❑ Yes ❑ No

	Very serious(5)	Serious(4)	Average (3)	Low (2)	Very low (1)
1.5.19.1 If Yes, what is the extent of such problem?					

1.5.19.2. How are these problems solved?

____

____

____

1.5.20. Was there any incident of an external government agency imposing its own rules on the community managing the forest?

❑ Yes ❑ No

	Strongly agree(5)	Agree(4)	Neutral (3)	Disagree (2)	Strongly disagree (1)
1.5.21. In general, the rights of users to devise their own institutions are not challenged by external governmental authorities.					

1.5.22. Can members of the community call a general meeting when they want to discuss a special problem such as violations (breaking) of community rules (by-laws) by members of the executive committee or general representative body?

❑ Yes ❑ No

1.5.22.1. If yes, has a special meeting been called during the last one years?

❑ Yes ❑ No

1.5.22.2. If no, has a special meeting been called during the last five years?

❑ Yes ❑ No

1.5.23. Has the executive committee or general representative body changed any rules of the forest user groupas a result of suggestions made by members?

❑ Yes ❑ No

1.5.24. Are there external organizations that are involved in the provision of training and technical assistance for community forest members?

❑ Yes ❑ No

1.5.24.1. If yes, please describe the external organizations that are involved in such activities.

____

____

____

____

____

____

1.5.25. Are there local organizations that are involved in the provision of training and technical assistance?

❑ Yes ❑ No

1.5.25.1. If yes, please describe the local organizations that are involved in such activities.

____

____

____

____

____

____

____

1.5.26. Are there any external organizations involved in management of the resource, i.e., in management of the community forest?

❑ Yes ❑ No

1.5.26.1. If yes, please describe external organization involved in management of the resource.

____
____
____
____
____
____
____

1.5.30Are there any other forest groups or CFUGs that exist in your community?

❑ Yes ❑ No

1.5.31. If yes, are similar rules, regulations, monitoring and sanctioning, etc. used?

❑ Yes ❑ No

1.5.32. If no, in what way are the rules, regulations, monitoring and sanctioning, etc. used or applied to these other CPR (s) different?

____
____
____

1.6. Additional Questions on Community Rules

NOTE TO ENUMERATORS: RULES AND REGULATIONS CAN BE INFORMAL AND NEED NOT BE TRULY LEGISLATED. PLEASE ENCOURAGE RESPONDENTS TO THINK BROADLY ABOUT THEIR RULES AND REGULATIONS

1.6. Does the community have any formal, informal or customaryrules and regulations that govern the access, use (harvesting), and maintenance (management) of the forest?

❑ Yes ❑ No

THE FOLLOWING QUESTIONS SHOULD BE ANSWERED ONLY IF THE COMMUNITY HAS SOME TYPES OF RULES AND REGULATIONS

1.6.1. Do these rules and regulation exist in writing?

(1) Yes, they are in written form. (2) No, they are oral ones

1.6.2. Are specific individuals in this community of users (user group) responsible for making the rules about the forest?

❑ Yes ❑ No

	Strongly agree(5)	Agree(4)	Neutral (3)	Disagree (2)	Strongly disagree (1)
1.6.3 Most individuals (members) affected by the operational rules of the community are included in the group that can modify rules					

1.6.4. Who enforces the rules and regulations (by-laws) that are created by the community? _______________

(1) By members of the community itself. (2) By officials (guards) selected by the community

(3) By external officials appointed by a government. (4) Others ways (describe) ____

1.6.5. If enforced by officials selected by the community, how does the community select officials (guards) for protection of the community forest/woodlot? What are the criteria to be selected? ____________.

(1) Decided in a meeting by the users (2) anybody interested can be a member of the officials (voluntary)

(3) Other (describe) ______________________

1.6 .6. On whom does the community enforce the rules and regulations? ________________ (multiple answers possible)

(1) On outsiders (non-beneficiaries) (2) Members of community (beneficiaries) (3) Others (specify) __________

1.6.7. How are the rules and regulations enforced? ___________

(1) Through patrolling (2) Others (specify) _____________________________

1.6.7.1. If through patrolling, explain the mechanism of patrolling?

____
____
____
____
____

1.6.7.2. If through patrolling such as by officials or guards, how often do you do the patrolling? __________

(1) Twice a day (2) Once a day (3) Every other day (4) Weekly (5) Others (specify) ________________

1.6.7.3. If through patrolling such as by officials or guards, what is the timing of the patrolling? _____________________

(1) Morning and evening (2) Every morning (3) Every evening (4) Others (specify) ________________

1.6.7.4. If through patrolling by members, how are they rewarded? ____

1.6.8. Can the community change the rules and regulations that govern the access, use and maintenance (management) of the forest resource whenever the need arises?

❑ Yes ❑ No

1.6.8.1. If yes, what is the procedure/mechanism involved to change these rules?

____
____
____
____

1.6.9. In your observation, do individuals in the community (user group) follow the rules and regulations for accessing and harvesting products from the forest or using the forest for non-consumptive purposes? _______________

(1) Rarely or never (2) Sometimes (3) About half the time (4) Most of the time

(5) Yes, almost always

1.6.10.Has there been any incident of an external government agent in one way or another using their authority to support those who refuse to follow the rules of the community managing or protecting the forest?

❑ Yes ❑ No

1.6.11Do you have any penalty system? ________________________

❑ Yes ❑ No

1.6.11.1. If yes, who decides what kind of penalty is appropriate when rule is observed to be violated or broken? _____

(1) The guard patrolling the community forest (2) A vote of individuals is the user group at a meeting
(3) A vote of an executive committee of the user group/community
(4) A vote of individuals in the user group/community and other authorized users of the forest/woodlot
(5) Others (describe) ____

1.6.11.2. If yes, cana cash or in-kind fine be imposed?
❑ Yes ❑ No

1.6.11.2.1. If yes, who collects the fines? ________________
(1) The guard who apprehends a rule breaker (2) An officials of the user group or community forest(3) The VDC (4) Others (describe) ____________

1.6.11.2.2. How is this fine used? ___________
(1) A general source of revenue for the community/members (2) A general source of revenue for local government

(3) A general source of revenue for forestry department (4) Others (describe) ___________________________

1.6.11.2.3. To what extent are these penalties complied with if imposed? ______________
(1) No one complies with the penalties imposed on them (2) Few users comply with the penalties imposed on them

(3) About half the users comply with the penalties imposed on them (4) Most users comply with the penalties imposed on them
(5) Penalties are fully complied with when imposed

1.6.11.2.4. If the users do not pay fines, what alternatives are open to the authority that imposes the fines? ___________
(1) Private reprimand and request to fully comply with penalty (2) Public reprimand and request to fully comply with penalty

(3) Confinement in local jail (4) Taking user to court (5) Others (describe) ___________________________________

	Strongly agree(5)	Agree(4)	Neutral (3)	Disagree (2)	Strongly disagree (1)
1.6.12. In general, users who violate operational rules are likely to receive sanctions or penalties that move from less serious to more serious penalties according to the severity of the violation.					

Section 2: Access on Service and Assets (Regarding forest resource survey and conservation)

2.0.1 Is there afforestation plan? ❑ Yes ❑ No

2.0.2 Does your FUG actively protect the forest ❑ Yes ❑ No

1.2 Information from forest operational plan

2.1a Is there a forest operational plan? ❑ Yes ❑ No

2.1b Does your FUG actively protect the forest ❑ Yes ❑ No

	Decreased (1)	No change (2)	Increased (3)
2.1.1 What types of changes do you observe in your forest condition in last 5 years in terms of biomass?			
2.1.2 What types of changes do you observe in your forest condition in last 5 years in terms of plant biodiversity?			
2.1.3 Do you think your forest have supported the local livelihoods?			

2.1.6 What changes have happened in the availability of forest products to present since youstarted to protect forest

SN	Types of forest products	Availability			Reasons
		Increased	Decreased	No change	
2.1.6.1	Timber				
2.1.6.2	Fuelwood				
2.1.6.3	Grass				
2.1.6.4	Leaflitter				
2.1.6.5	Green leaves for animal bedding				
2.1.6.6	Agricultural implements				
2.1.6.7	NTFPs				
2.1.6.8	Others				

0.7 What changes have forest management activities brought in the condition of forest to FY 2012 since you started to protect theforest?

S.N.	Areas of change	Increased	Decreased	No change	Reason	
2.1.7.1	Tree covered areas					
2.1.7.2	Density of forest					
2.1.7.3	Species					
2.1.7.4	Productivity of forest					
2.1.7.5	Regeneration					
2.1.7.6	Bio-diversity					
2.1.7.7	Others					

Section 3: Access to Services/Assets by Forest User Group/Forest Protection Committee Members

Fill the following information from the account and other records of FUG IF AVAILABLE. OTHERWISE LEAVE BLANK

3.1 Income

Review the income ledger of FUG, if any and fill the following information in NRs. by income headings and source.

SN	Income headings	Total income in NRs.		
		Fiscal year 2010	Fiscal year 2011	Fiscal year2012
1	Carry forward from previous year			
1.1	Bank balance			
1.2	Cash balance/advance/deficit			
1.3	Loan investment for income			
1.4	Other loan investment			
	Total			
2	Wood/timber selling			
3	Firewood selling			
4	Pole selling			
5	Agriculture tools selling			
6	Grass and green leaves selling			
7	Non-timber forest product selling			
8	Grants (Local bodies)			
9	Other grants			
10	Loan (taken by FUG)			
11	Penalties/fine			
12	Interest			
13	Advance not cleared			
14	Research service fee			
15	Membership/annual/renewal fee			
16	Entry fee			
17	Program support			
18	Others			
	Total			
	Grand total			

3.2 Expenditure

SN	Topic	In NRS		
		Fiscal year 2010/2011	Fiscal year 2011/2012	Fiscal year 2012/2013
	Community development			
	Community services			
	Enterprise development program			
	Income generating			
	Training, workshop and study tours			
	Administrative expenses			
	Interest, donation, grants			
	Current year balance			
	Grand total			

3.4 Production and selling of forest products (last fiscal year –i.e. 2011/12)

Review the 'ProductIon and sale of forest products' ledger or by asking, if relevant, and fill the following table

S. N.	Type of forest product	Unit	Total production	Internal distribution and selling					Outside selling	
				Free of cost	Paid					
					Subsidized price		Full cost			
				Qty	Qty	Amount NRs.	Qty	Amount NRs.	Qty	Income
1	Timber	Cu ft.								
2	Firewood	Bhari								
3	Pole	Pieces								
4	Agri. tools	Number								
5	Grass	Bhari								
6	Fodder	Bhari								
7	Dry leaves	Bhari								
8	Non-timber									
8.1										
8.2										
8.3										
8.4										
9	Others									

3.7 Other Issues Added

	Strongly agree(5)	Agree(4)	Neutral (3)	Disagree (2)	Strongly disagree (1)

3.7.2. Rules specifying the amount of forest product(s) that a user or member is allocated are related to their labor, materials and/or money inputs.					
3.7.3. Those (members) who receive the highest proportion of the resource are required to pay the highest proportion of the costs.					
3.7.4. Rules to apportion benefits and costs take into account unique features of the community forest (local conditions).					
3.7.5. The system for deciding who has access to the forest resources is a fair one.					
3.7.6. Benefits are distributed fairly.					

3.7.7. What mechanisms do you use to distribute the benefit from the forest? ____________
(1) Harvest in-group, divide harvest into equal lots and distribute(2) Individually harvest products or graze animals
(3) Harvest in group, divide harvest into lots based on parameters & distribute accordingly (4) Others (describe) _______________

3.7.8. Have there been any change(s) over time in the mechanism used to distribute benefits from the forest?
❑ Yes ❑ No

3.7.8.1. If yes, what was/were the reason(s) for the change in the mechanism used in allocating or distributing benefits? _______________

3.7.9. Are there problems (complaints) related to the distribution of benefits of the forest?
❑ Yes ❑ No

3.7.9.1. If yes, what are the problems related to the distribution of benefits?

3.7.10. What are the contributions expected of members?
(1) Labor (2) Money (3) Others (specify)___________________________ (4) None

3.7.11. How does the community perceive itself in terms of relating to other forest-governing structures?
(1) Not cooperating

(2) Cooperating, but independent of other organizations rules and regulations

(3) Cooperating jointly in determining rules and regulations

Section 4: Capacity building and empowerment

4.1.10 What are the processes for comittee meetings and decision making?

Activities performed by FUG/FPC	Who does it?	Activities performed by general assembly	Who does it?
Deciding date and venue		Deciding date and venue	
Setting the agenda?		Setting the agenda?	
Communicating users, committee members		Communicating users, committee members	
Making decisions		Making decisions	

4.3 Public audit and public hearing

4.3.1 Is there any practice of public audit? ❑Yes ❑No

4.3.2 Is there any practice of public hearing? ❑ Yes ❑ No

4.5 Annual report and financial audit

4.5.1. Is financial audit completed? ❑Yes ❑No (If no, why ....)

Section 5: Social inclusion: Practical changes in policy, social norms and values

4.6 Constitution and operational plan

4.6.1 Provision for poor and excluded groups in FUG's constitution and/or operational plan (if applicable)

S.N.	Provision in constitution and operational plan FUG	Status
1	Provision of subsidy or no fee for poor and excluded groups in selling and distribution of forest product	❑Yes ❑No
2	Provision of fund mobilization for the welfare of women, poor and excluded HHs	
	a) in health	❑ Yes ❑ No
	b) in education	❑ Yes ❑ No
	c) in income generation and enterprise development	❑ Yes ❑ No
	d) others, specify	❑ Yes ❑ No
3	Provision of creating employment for women, poor and excluded HHs	
	a) daily wages	❑ Yes ❑ No
	b) employment	❑ Yes ❑ No
4	Provision of cash allowance for women, poor and excluded HHs while attending meetings or participating training and/or development works	❑ Yes ❑ No
5	Provision of giving priority to women, poor and excluded HHs in training, workshop and visits	❑ Yes ❑ No
6	Provision of representation of women, poor and excluded HHs in vital posts of executive committee	❑ Yes ❑ No
7	Provision of forest land allocation to generate income of women, poor and excluded	❑ Yes ❑ No

4.6.2 Decisions made by FUG.

Program	Who decides?		
	Committee	Group member/assembly	Both
Determine forest area for protection			
Policy for proper utilization of forest products			
Choosing forest protection methods			
Decide provision on penalties			
Implement penalty provisions			
Develop mechanisms for benefit sharing and forest products distribution			
Develop forest operational plan			
Select plants and trees			

Nursery management			
Plantation			
Management of group income			
Decide training/visits			
Determine income generating activities			
Determined community development program			
Other program (specify)			

4.6.3 How did the FUG establish mechanisms to distribute firewood and grass?

❑ Adapted from other FUG ❑Advice from forest technician

❑By group itself ❑By committee

0.22How do you assess service received from government forest technician? ❑very good ❑good ❑okay ❑ Bad

0.23 What is the reason for the abovesaid assessment?

❑ Provided service without hassle ❑Provided service with hassle

❑ Provided required information

❑ required information not provided ❑Others (specify)

0.24 What are the mechanisms and processes to promote good governance (transparency, accountability, inclusive, participatory, and rule of law)?

❑ Public audit ❑Public hearing ❑Others (specify)

❑ have not done anything

0.25 What are the processes followed by FUG to promote good governance?

❑

0.26Are there any provisions to address gender and social inclusion issues in the budgeting process?

❑ Yes❑ No

0.26.1 If yes, specify.

❑

0.29 Is there any provision to include poor and excluded groups while developing policy and strategies?

❑ Yes❑ No

0.30 If yes, specify.

❑

0.31 How are annual programmes prepared?

❑ Prepared in general assembly ❑Prepared and implemented by executive committee ❑Rangepost

❑Draft prepared by executive and approved by general assembly ❑ Others

0.32Details of employees hired by FUG

Employee's	Designation	Dalit	Janajati	Brahmin/Chhetri	Madhesi	Muslim	Others	Remarks

Name		Hill		Madhesi		Others				Newar		Hill		Madhesi								(wellbeing class)
						Hill		Madhesi														
		F	M	F	M	F	M	F	M	F	M	F	M	F	M	F	M	F	M	F	M	

0.36 How many times executive committee meetings occurred in last two years? : .... times

5.4 Rule compliance and punishment

5.4.1 What is the trend of forest product theft frequency? ❑Increased ❑Decreased ❑ No change

5.4.2 Generally, who is involved in these crimes, and why? ❑Male ❑Female ❑ Both

5.4.3 Generally, who is involved in these crimes? ❑Villagers ❑Outsiders ❑ Both

5.4.4 Generally, which kind of forest resources are stolen? ❑Firewood ❑Grass❑ Small logs❑ Large logs❑ NTFPs

5.4.8 How much cubic feet of timber was decided to be harvested annually? ....

5.4.9 What is the annual allowable harvest (AAH) of timber ……………….cu. ft.*(If applicable, otherwise leave blank)*

5.4.10 Has a higher amount of timber than the decision (or AAH) been harvested? ❑ Yes ❑ No

5.4.11 Specify the reasons, if more trees are harvested than annual allowable harvest

❑

5.4.12What kind of trees have been harvested?

❑ fallen ❑ dried ❑ old and matured

❑ green ❑ suffered from insect and pest ❑ other

5.4.18 On the basis of experience, what are the positive aspects and challenges of FUG?

Positive aspects	Negative aspects

5.4.19 What are the good practices in FUG in addressing issues of women, poor and excluded groups; enhancing good governance; and improving forest quality?

Addressing issues of women, poor and excluded groups	Enhancing good governance	Improving forest quality

5.4.20 What are the areas of improvement in FUG to address issues of women, poor and excluded groups; enhance good governance; and improve forest quality?

Addressing issues of women, poor and excluded groups	Enhancing good governance	Improving forest quality

Please write your notes or suggestions, if any.

Thank you

For internal purpose only

Participating community members

SN	Name	Designation	Telephone number	
			Home	Mobile

Information collected by:

Designation:

Date:

Signature:

Checked by:

Designation:

Date:

Signature:

Carbon Stock Measurement Form (CSMF)

Plot No.: ____________

Strata: ____________

District:

Measurement started at: ____________ (time, e.g., hour : minute)

Date: / /2010 (dd/mm/yy)

Team leader: ____________

Team members: ____________

Rough sketch showing the plot:

References for the plot center:

1. Background information

CFUG Name: ____________

Forest Name: ____________

Block number: ____________

**GPS co-ordinates**		
	UTM-X	m
	UTM-Y	m
	Altitude	m

2. Plot information

	Please circle one
Forest type:	natural / plantation
Aspect:	N, S, E, W, NE, NW, SE, SW, Flat
Soil type:	clayey, loam, sandy, boulder
Fire:	Yes / No
Fodder collection:	Yes / No
Grazing:	Yes / No
Fuelwood collection:	Yes / No
Timber harvesting:	Yes / No
Encroachment:	Yes / No
Wildlife:	Yes / No
Soil erosion:	Yes / No

Please give a brief reason why if the plot has been relocated from the originally given GPS position.

Vegetation type:	
Slope:	degree (average)
Soil color:	
Soil depth:	m
Crown cover:	%
Shrub cover:	%
Grass cover:	%

Any additional information

Tree-DBH and height measurement within **8.92m** radius horizontal distance.

CF( )/ Non CF( ) Plot No…………

Note: For clinometer with 15/20 reading use tree DBH and height column only.

S. N	Species	DBH (cm) @ 1.3 m	Angles formed by top and base of the tree		Distance to the tree(m) (D)	Slope condition	Tree Height (M)	Remarks
			Top (A)	Base (B)				

All trees within the plot with DBH≥5 cm have been measured.
The species of unidentified trees have been recorded as Sp 1…..Sp 2 likewise and distinguishable characteristics are noted as comment.

Tree-DBH and height measurement within **8.92m** radius horizontal distance.

CF(   )/ Non CF(   )   Plot No…………

Note: For clinometer with 15/20 reading use tree DBH and height column only.

**S. N**	**Species**	**DBH (cm) @ 1.3 m**	**Angles formed by top and base of the tree**		**Distance to the tree(m) (D)**	**Slope condition**	**Tree Height (M)**	**Remarks**
			Top (A)	Base (B)				

All trees within the plot with DBH≥5 cm have been measured.
The species of unidentified trees have been recorded as Sp 1…..Sp 2 likewise and distinguishable characteristics are noted as comment.

Sapling (1 cm to 5 cm)- measure within **5.64 m** circular plot

District………………………………………………… CF(   )/Non CF(   )   Plot No…………

S.N	Species	DBH (cm)	S.N	Species	DBH (cm)
1			14		
2			15		
3			16		
4			17		
5			18		
6			19		
7			20		
8			21		
9			22		
10			23		
11			24		
12			25		
13			26		

Regeneration (below 1 cm DBH)- measure within a **1 m** circular plot

District………………………………………………… CF(   )/Non CF(   )   Plot No…………

S.N	Species	Total Count	S.N	Species	Total Count
1			6		
2			7		
3			8		
4			9		
5			10		

Tools and goods

GPS……….., Santu clinometer……………, Calculator……………,

Measuring tape ………………… D-Tape……….. Other stationary goods……………

Form filled by:

Name…………………………………………………. Signature………………………………………………………………
